# Long chain acyl CoA synthetase 4 catalyzes the first step in peroxisomal indole-3-butyric acid to IAA conversion

**DOI:** 10.1093/plphys/kiaa002

**Published:** 2020-11-17

**Authors:** Vanessica Jawahir, Bethany Karlin Zolman

**Affiliations:** Department of Biology, University of Missouri – St Louis, St Louis, Missouri 63121, USA

## Abstract

Indole-3-butyric acid (IBA) is an endogenous storage auxin important for maintaining appropriate indole-3-acetic acid (IAA) levels, thereby influencingprimary root elongation and lateral root development. IBA is metabolized into free IAA in peroxisomes in a multistep process similar to fatty acid β-oxidation. We identified LONG CHAIN ACYL-COA SYNTHETASE 4 (LACS4) in a screen for enhanced IBA resistance in primary root elongation in *Arabidopsis thaliana*. LACSs activate substrates by catalyzing the addition of CoA, the necessary first step for fatty acids to participate in β-oxidation or other metabolic pathways. Here, we describe the novel role of LACS4 in hormone metabolism and postulate that LACS4 catalyzes the addition of CoA onto IBA, the first step in its β-oxidation. *lacs4* is resistant to the effects of IBA in primary root elongation and dark-grown hypocotyl elongation, and has reduced lateral root density. *lacs6* also is resistant to IBA, although both *lacs4* and *lacs6* remain sensitive to IAA in primary root elongation, demonstrating that auxin responses are intact. LACS4 has in vitro enzymatic activity on IBA, but not IAA or IAA conjugates, and disruption of LACS4 activity reduces the amount of IBA-derived IAA *in planta*. We conclude that, in addition to activity on fatty acids, LACS4 and LACS6 also catalyze the addition of CoA onto IBA, the first step in IBA metabolism and a necessary step in generating IBA-derived IAA.

## Teaser

An enhancer mutant revealed an acyl-CoA synthetase that catalyzes CoA addition to indole-3-butryic acid, required for the β-oxidation steps necessary to generate indole-3-butryic acid-derived IAA.

## Introduction

Auxin controls plant cell elongation, division, and differentiation and is, therefore, crucial for development throughout the life span of a plant. Because of its diverse and extensive effects, proper levels of indole-3-acetic acid (IAA), the primary signaling auxin, are tightly controlled through synthesis, degradation, transport, and sequestration (Korasick et al., 2013). Auxin can be sequestered in inactive forms by conjugation to amino acids or side chain elongation to form indole-3-butyric acid (IBA). IAA conjugates are converted back to IAA with a single hydrolysis reaction (Korasick et al., 2013). IBA is structurally similar to IAA but the side chain is two carbons longer; IBA is metabolized to IAA in the peroxisome in a multistep process (Hu et al., 2012). These unique input pathways vary between individual cells, organs, and developmental stages but all function to ensure proper levels of this crucial hormone.

IBA**-**derived auxin influences cotyledon expansion, apical hook formation, and several aspects of root architecture, including primary root elongation, lateral root development, root hair elongation, and adventitious root development (Korasick et al., 2013; Frick and Strader, 2018). Evidence suggests that IBA to IAA metabolism in the root tip is the primary source of auxin to initiate lateral root development (De Rybel et al., 2012; Xuan et al., 2015) and reinforces the auxin gradient to elongate lateral roots (Strader and Bartel, 2011). Recent evidence suggests IBA β-oxidation is required for root hair elongation under low-phosphate conditions, connecting IBA effects to changing environmental conditions (Trujillo-Hernandez et al., 2020). Such developmental roles are hypothesized to be due to the actions of IBA-derived IAA instead of direct effects of IBA. Analysis of IBA-response (*ibr*) mutants suggests IBA is β-oxidized into IAA in a stepwise process paralleling fatty acid β-oxidation, the process in which two carbons are removed from fatty acids (Hu et al., 2012).

In plants, the peroxisome is the sole location of fatty acid β-oxidation. Peroxisomes house enzymes for three crucial β-oxidation pathways: fatty acid β-oxidation, jasmonic acid (JA) synthesis, and IBA conversion to IAA (Hu et al., 2012; Kao et al., 2018). Peroxisomes must actively import necessary enzymes and substrates required for these pathways. Substrates for β-oxidation are imported into peroxisomes through PEROXISOMAL ABC TRANSPORTER PXA1/PED3/CTS (Zolman et al., 2001; Hayashi et al., 2002). These molecules are broken down into two carbons per cycle.

Following fatty acid import, fatty acid β-oxidation begins with CoA addition by acyl-CoA synthetases LACS6 and LACS7 (Fulda, 2004). CoA addition is necessary to activate the fatty acid for recognition before metabolism proceeds. Fatty acyl-CoA esters are oxidized by acyl-CoA oxidases, hydrated and then dehydrogenated by the multifunctional proteins ABNORMAL INFLORESCENCE MERISTEM 1 (AIM1) and MULTIFUNCTIONAL PROTEIN 2 (MFP2), and cleaved by the 3-ketoacyl-CoA thiolases KAT2/PED1 and KAT5 to release acetyl-CoA and a shortened fatty acid (Graham, 2008; Hu et al., 2012). This process can be repeated as needed. For instance, very long-chain fatty acids, as found in seed storage lipids, enter the pathway multiple times to release many molecules of acetyl CoA. Acetyl-CoA can then enter the citric acid cycle to ultimately be oxidized for energy production (Hu et al., 2012). Peroxisomes are, therefore, crucial for early development and seedling establishment because plants rely on the energy produced by the breakdown of fatty acids stored as triacylglycerol before they are photosynthetically active (Graham, 2008). Plants with severe defects in fatty acid β-oxidation are compromised in their ability to generate the energy necessary for germination and growth for early seedling establishment. Defects in fatty acid β-oxidation can be exposed by examining growth on media without sucrose, which requires metabolism of seed storage lipids. Addition of sucrose to the media bypasses this requirement, allowing development to proceed. Mutants that require exogenous sucrose for normal growth are designated as sucrose dependent.

In a similar process, JA is the product of three rounds of β-oxidation of 12-oxo-phytodienoic acid, by the actions of ACX1, ACX5, AIM1, and PED1/KAT2 (Hu et al., 2012; Wasternack and Hause, 2013). The role of peroxisomes in JA synthesis makes them important for JA effects including fertility and response to abiotic and biotic stressors (Wasternack and Hause, 2013).

Peroxisomes also contribute to the pool of free IAA by β-oxidizing IBA. IBA is hypothesized to undergo one round of β-oxidation to remove the two-carbon elongation, producing IAA. Forward genetic screens have revealed proteins that are acting in IBA metabolism. *ibr3*, *ibr10*, and *ibr1* were identified in *Arabidopsis thaliana* as resistant to the inhibitory effects of IBA on root elongation. The predicted protein functions of these peroxisomal enzymes suggest that the process of IBA β-oxidation is mechanistically related to fatty acid metabolism, with the action of IBR3, a predicted oxidase, IBR10, a predicted hydratase, and IBR1, a predicted dehydrogenase, to release IAA (Zolman et al., 2007, 2008). IBR3, IBR10, and IBR1 appear specific to IBA metabolism as mutants do not have phenotypes associated with defects in fatty acid β-oxidation, JA synthesis, or peroxisomal import (Zolman et al., 2007, 2008). Mutations to known β-oxidation enzymes, including *aim1* and *ped1*, have IBA-response phenotypes as well (Hayashi et al., 1998; Zolman et al., 2000); these phenotypes could indicate redundant enzymatic activity, with multiple enzymes acting on IBA at each step, or enzyme activity on multiple substrates affecting both pathways. Alternatively, IBA-resistant responses in the mutants *acx1*, *acx3*, *chy1*, and *ech2* suggest that indirect mechanisms, such as CoA limitations (Adham et al., 2005) or accumulation of toxic intermediates (Zolman, Monroe-Augustus, et al., 2001; Li et al., 2019), also influence peroxisomal metabolic pathways.

IBA metabolism requires that the peroxisome must actively import functional proteins, substrates, and cofactors necessary for the contained metabolic processes (Hu et al., 2012; Kao et al., 2018). Enzymes destined for the peroxisome matrix typically contain one of two peroxisome targeting signals (PTSs): PTS1, a C-terminal tripeptide with the consensus amino acids [S][RK][LM], or PTS2, a R[LI]X_5_HL near the N-terminus (Reumann, 2004; Lanyon-Hogg et al., 2010). IBA-resistant phenotypes can be caused by mutations specific to IBA metabolism, such as defects in IBA-specific enzymes or by disrupting function of the organelle as a whole. For instance, mutations in PEX5 and PEX7, receptors required for import of enzymes into the peroxisome matrix (Zolman et al., 2000; Woodward and Bartel, 2005; Khan and Zolman, 2010) and the PXA1/CTS/PED3 (Zolman et al., 2001) peroxisomal substrate transporter each disrupt IBA responses.

Although many components specific to IBA metabolism and peroxisome function have been identified, our understanding is incomplete. For instance, the CoA synthetase initiating IBA β-oxidation and a peroxisomal IAA efflux carrier have not been identified (Hu et al., 2012; Damodaran and Strader, 2019). We sought to identify additional components of IBA metabolism by continuing the forward genetic screen for IBA-resistant plants. In this study, we describe our identification of long-chain CoA synthetase 4 (LACS4) from an enhancer screen using an *ibr3* mutant background. The mutant phenotypes and enzymatic activity of LACS4 suggest that it functions as the initial step in IBA metabolism, charging IBA with CoA allowing entry into the β-oxidation process. This discovery completes the enzymatic components that metabolize IBA to IAA.

## Results

### Isolation of mutants with enhanced IBA resistance

We conducted a screen to identify *Arabidopsis* mutants with altered responses to IBA in root development. To increase our chances of finding novel components, an enhancer screen was performed. *ibr3* is resistant to both the inhibition of IBA on root elongation and the stimulatory effect of IBA on lateral root initiation (Zolman et al., 2007). *ibr3* was chosen as the background because it displays weaker IBA resistance than other *ibr* mutants (Zolman et al., 2008), facilitating the potential to capture a range of enhancement phenotypes.

*ibr3-1* seeds were treated with ethylmethane sulfonate (EMS) to induce point mutations. Mutagenized populations were screened for seedlings exhibiting enhanced resistance to IBA in root length. This phenotype parallels the original screen (Zolman et al. 2000) and is facile relative to lateral root or root hair phenotypes. We hypothesized we could identify factors involved in the function or regulation of IBR3, the conversion of IBA to IAA, and/or general peroxisome function in Arabidopsis.

### *Z377* is defective in several IBA responses

**Figure 1:**
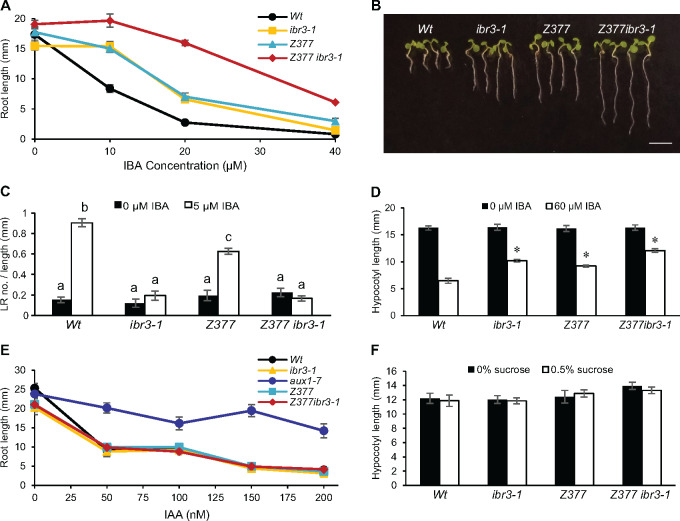
*Z377* has phenotypes specific to disruptions in IBA metabolism. (**A**) Primary root elongation of 7-d-old seedlings grown on increasing concentrations of IBA (± se, *n* ≥10). (**B**) Images of 7-d-old seedlings grown on 20 μM IBA. Scale bar = 5mm. (**C**) Lateral root density of 8-d-old seedlings was quantified by dividing the number of lateral roots by the primary root length. Statistical significance was determined by two-way ANOVA with *post hoc* Tukey HSD (± se, *n* ≥8, *P* < 0.001). Common letters indicate no significant difference. (**D**) Length of dark-grown hypocotyls. Seedlings were grown for 1 d in light and 5 d in darkness. Statistical significance was determined by two-tailed *t* test versus *Wt* on the same condition (± se, *n* ≥ 15, *P < 0.001). (**E**) Primary root length of 7-d-old seedlings grown on increasing concentrations of IAA (± se, *n* ≥ 8). (**F**) Hypocotyl length of seedlings grown on media with and without sucrose for 1 d in light and 5 d in darkness. Statistical significance determined by one-tailed *t* test between sucrose treatments (± se, *n* ≥ 13, *P* <0.05).

Exogenous IBA inhibits root elongation of wild-type (Wt) plants and *ibr3-1* at higher concentrations. One enhancer mutant, *Z377 ibr3-1*, was selected for further characterization based on its increased resistance to IBA in root elongation (Figure 1A). Wild-type plants grown on 20 *µ*M IBA have a primary root length <20% of that when grown without hormone supplementation (Figure 1, A and B). *ibr3-1* is double that with a primary root length of 40% the root length without hormone. In stark contrast, *Z377 ibr3-1* retains a root length of >80% on the same IBA concentration.

Following its confirmation as an *ibr3* enhancer in root elongation, *Z377 ibr3-1* was examined for defects in lateral root initiation. IBA is effective at inducing lateral rooting; a wild-type plant will develop branched root architecture when grown in the presence of IBA compared to an elongated, less branched root in the absence of hormone (Zolman et al., 2000). Decreased induction of secondary roots is a prominent phenotype in many IBA-resistant mutants, including *ibr3-1* (Zolman et al., 2000, 2007). Lateral root development can be quantified by counting the number of lateral roots that develop per millimeter along the primary root. *Z377 ibr3-1* is defective in lateral root initiation when stimulated with IBA, resembling wild-type grown in the absence of hormone (Figure 1C). This phenotype is similar to the *ibr3-1* parent line.

IBA has prominent effects on root development but also affects shoot development (Strader et al., 2011; Frick and Strader, 2018). To determine if the IBA-response defects of *Z377 ibr3-1* are specific to roots or affected throughout the plant, hypocotyl elongation was measured as an indicator. Wild-type plants have reduced hypocotyl length when grown on IBA than media without hormone (Figure 1D). The enhanced resistance of *Z377 ibr3-1* to IBA extends to dark-grown hypocotyl elongation, indicating the IBA-response defects are not limited to specific tissues.

To understand the mutation leading to these enhanced responses, the *Z377* single mutant was separated from *ibr3-1* following a backcross to wild-type. F_2_ progeny were characterized at a genotypic and phenotypic level. IBA-resistant individuals were selected, then tested to determine their genotype at the *IBR3* locus. A subset of lines was identified that showed elongated roots on IBA but a wild-type *IBR3* genotype. These lines were hypothesized to represent the effects of the *Z377* mutation alone. These mutants had IBA resistance in root elongation assays intermediate between wild-type seedlings and the *Z377 ibr3-1* enhancer and were comparable to the single *ibr3* mutation (Figure 1, A and B). Surprisingly, *Z377* remained sensitive to IBA-induced lateral rooting (Figure 1C). *Z377* develops a similar mean number of lateral roots to wild-type, with somewhat reduced lateral root density because of its longer primary root. This phenotype is distinct from *ibr3*, which has statistically similar lateral root density when grown with or without IBA (Figure 1C), and other *ibr* mutants (Zolman et al., 2008). Additional backcrosses revealed that the *Z377* mutation segregates in a recessive manner.

### *Z377* phenotypes are specific to IBA

To further understand the range of *Z377*-related phenotypes, we tested other common responses related to IBA metabolism using the single mutant and enhancer line. First, *Z377* and *Z377 ibr3-1* were tested for responses to IAA to determine if these mutants had IBA-specific defects or a generalized resistance to auxin. *Z377* and *Z377 ibr3-1* remained sensitive to inhibition of primary root elongation by IAA (Figure 1E).

Enzymatic components of the IBA metabolic pathway discovered to date are contained within the peroxisome and several mutants with fatty acid β-oxidation defects also have IBA-resistant phenotypes (Hayashi et al., 1998; Zolman et al., 2000; Adham et al., 2005). To examine if *Z377* is involved in fatty acid β-oxidation or peroxisomal functions, *Z377* was tested for sucrose-dependent growth. Both *Z377* and *Z377 ibr3-1* have comparable elongation of dark-grown hypocotyls in media lacking or containing sucrose (Figure 1F).

These initial experiments suggest that the causative mutation in *Z377* specifically disrupts IBA metabolism, but not fatty acid β-oxidation, and the mutants have normally functioning peroxisomes.

### *Z377* is defective in LACS4

**Figure 2:**
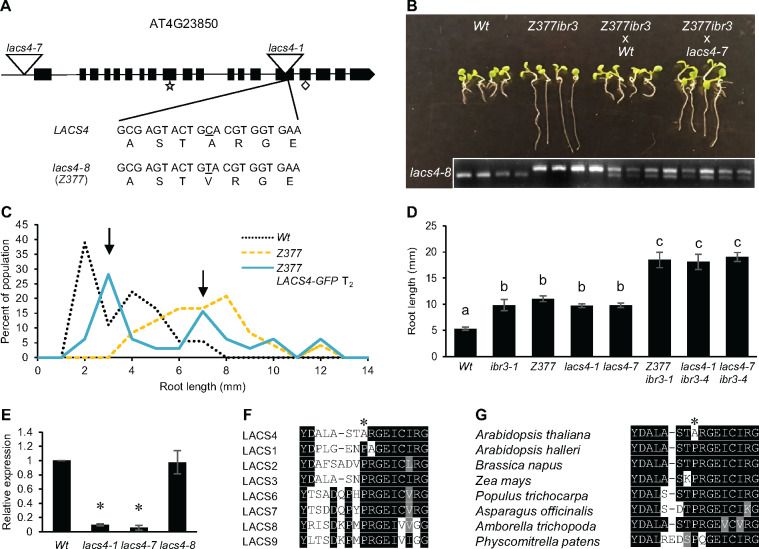
*Z377* is defective in *lacs4*. (**A**) Schematic of *LACS4* gene structure. Exons are represented as black boxes whereas introns are lines. Locations of T-DNA insertion mutants used in this study are depicted as inverted triangles. The mutation in *Z377* changes nucleotide 1,400 from C to T, resulting in an alanine to valine substitution at residue 467. As a result, Z377 was renamed as *lacs4-8*. Predicted AMP and fatty acid binding motifs (UniProt Consortium, 2019) are indicated by a star and diamond, respectively. (**B**) *Z377 ibr3-1* was crossed to *Wt* and *lacs4-7* in a test of non-complementation. F_1_ seedlings were grown on 20 *µ*M IBA for 7 d. Seedlings were genotyped for the point mutation in *LACS4* in *Z377* to confirm the cross was successful. The *Wt LACS4* allele is the smaller product whereas the mutant *lacs4* is the larger product. (**C**) *Z377* seedlings segregating a *Wt* copy of *LACS4* tagged with a C-terminal GFP were grown on 15 *µ*M IBA for 7 d. Length of primary root was measured for each individual. Data are represented as percent of the population with indicated primary root length (*n* ≥ 18 *Wt* and *Z377*, *n* = 32 *Z377* 35S: *LACS4*-GFP). (**D**) Primary root elongation of 7-d-old seedlings grown on 20 *µ*M IBA. Statistical significance was determined by one-way ANOVA with *post hoc* Tukey HSD test (±SE, *n* ≥ 11, *P* < 0.05). Common letters indicate no significant difference. (**E**) Expression of *LACS4* relative to *Wt* in 5-d-old seedlings grown under white light on filter paper. *LACS4* expression is normalized against *UBQ10.* Statistical significance was determined by a two-tailed *t* test (± se, *n* = 5, *P < 0.001). (**F**) and (**G**) T-Coffee alignment of LACS proteins of *A. thaliana* (**F**) and LACS4 orthologs in divergent plant species (**G**). Residues depicted are 460–475 of LACS4 from *A. thaliana.* The amino acid residue mutated in *lacs4-8* is indicated with an asterisk. Black shading indicates the same amino acid. Gray shading indicates similar amino acids.

Whole-genome sequencing was used to identify the causative mutation in *Z377*. DNA extracted from 10 independently isolated *Z377 ibr3-1* lines from a backcross to *ibr3-1* was pooled for sequencing. Parallel experiments were done for wild-type and *ibr3-1*. Gene candidates were first narrowed by identifying homozygous single nucleotide polymorphisms consistent with EMS mutagenesis in *Z377 ibr3-1.* Mutations common between *Z377 ibr3-1* and wild-type or *Z377 ibr3-1* and *ibr3-1* were eliminated, leaving 10 mutations unique to *Z377 ibr3-1.* Nine candidate genes were clustered on chromosome 4 (Supplementary Table S1). *AT4G23850*, which encodes LACS4, showed an alanine to valine substitution in the *Z377* mutant (Figure 2A) and became the prime candidate because LACS6 and LACS7 are known peroxisomal enzymes responsible for priming fatty acids with CoA in fatty acid β-oxidation (Fulda, 2004). We hypothesized that LACS4 may be the CoA synthetase responsible for catalyzing the addition of CoA onto IBA as this enzyme has not yet been identified (Hu et al., 2012). Other candidate genes did not have a clear link to peroxisomes, auxin responses, IBA metabolism, or lipid metabolism, further supporting the hypothesis that the mutation in LACS4 might be the causative mutation in *Z377.*

To test our findings in an unbiased manner, T-DNA mutants disrupting each candidate gene were acquired and screened for IBA resistance in primary root elongation (Supplementary Table S1). Only Salk_120357 showed an IBA-resistant response in root elongation assays like *Z377* (Supplemental Figure S1A). Salk_120357 is inserted in the 5′–untranslated region of *LACS4* (Figure 2A). Because several T-DNA alleles of *lacs4* were recently named (Zhao et al., 2019), we named this previously uncharacterized Salk line *lacs4-7.* In addition to resistance in primary root elongation, *lacs4-7* shows partial sensitivity to induction of lateral root formation, another phenotype consistent with *Z377* (Supplementary Figure S1B).

To confirm causality, we completed non-complementation testing for *Z377 ibr3-1*. F_1_ seedlings of *Z377 ibr3-1* backcrossed to wild-type were sensitive to IBA (Figure 2B), indicating that the mutation is recessive. We then crossed *Z377 ibr3-1* with *lacs4-7.* F_1_ progeny of this cross had intermediate resistance to IBA in primary root elongation, similar to *Z377* alone, supporting that the mutation in *LACS4* is causative.

Separately, we generated a complementation line of the *Z377* mutant transformed with the *LACS4* coding sequence. *Z377* lines segregating a 35S:*LACS4* transgene containing a green fluorescent protein (GFP) tag were analyzed to observe if IBA responses were recovered by the presence of a wild-type copy of *LACS4*. Primary root length of segregating lines grown on 15 *µ*M IBA was measured and scored for rescue relative to the IBA-resistant phenotype of *Z377*. The primary root IBA-resistant phenotype of *Z377* is successfully complemented by a *LACS4* construct, as indicated by the dual peaks representing the populations of rescued and nonrescued phenotypes within this segregating line (Figure 2C). The large peak with IBA-sensitive plants has root lengths less than the average root length of *Z377*, which suggests the addition of a wild-type *LACS4* copy restores wild-type to IBA in primary root elongation. The smaller peak of the segregating population shows plants with elongated roots, with growth consistent to the *Z377* mutant.

Finally, we demonstrated causation by generating novel allele combinations of *lacs4* and *ibr3* and looking for similar enhanced IBA-resistant responses. For this assay, we used *lacs4-7* and also expanded our analysis to include *lacs4-1*, a T-DNA allele found in an exon (Figure 2A) that has been previously described (Jessen et al., 2011, 2015; Zhao et al., 2019). We crossed *lacs4-1* and *lacs4-7* separately with a T-DNA line disrupting *IBR3* (Zolman et al., 2007)*. lacs4-1 ibr3-4* and *lacs4-7 ibr3-4* have enhanced resistance to IBA in primary root elongation and indeed recapitulated the phenotype seen in the original *Z377 ibr3-1* line (Figure 2D). Following these tests, we concluded that the mutation in *LACS4* is causative for the IBA-response defects of *Z377*. The *Z377* mutant is now named *lacs4-8* (Figure 2A).

### Missense mutation at residue 467 disrupts LACS4 function in IBA metabolism

The *lacs4* mutation in *Z377* is a substitution of an alanine to a valine at residue 467 (Figure 2A). Despite being what is traditionally defined as a weak mutation, *lacs4-8* yields IBA-resistant phenotypes as strong as each T-DNA allele (Figure 2D). To determine if *lacs4-8* disrupts function by altered expression or protein activity, expression of *LACS4* was tested in all three mutants using gene-specific primers. We found that *LACS4* is expressed in *lacs4-8* at wild-type levels, whereas *lacs4-1* and *lacs4-7* have dramatically reduced expression (Figure 2E), as previously noted for *lacs4-1* (Jessen et al., 2015)*.* This result suggests the mutation in *lacs4-8* results in translation of a nonfunctional LACS4 protein, which is as compromised in IBA metabolism as much as plants lacking LACS4 completely.

In *A. thaliana* LACS4, residue 467 encodes an alanine. Interestingly, alignment of the LACS4 protein sequence with the Arabidopsis LACS proteins revealed that residue 467 is a conserved proline in all other family members (Figure 2F). We expanded our inquiry to LACS4 orthologs in diverse species and discovered that this residue again is a completely conserved proline, even in the close relative *Arabidopsis halleri* (Figure 2G). This strong conservation suggests this alanine residue is specific for *A. thaliana* LACS4 function and may be a site sensitive to perturbations.

### *lacs4* and *lacs6* are resistant to IBA

**Figure 3:**
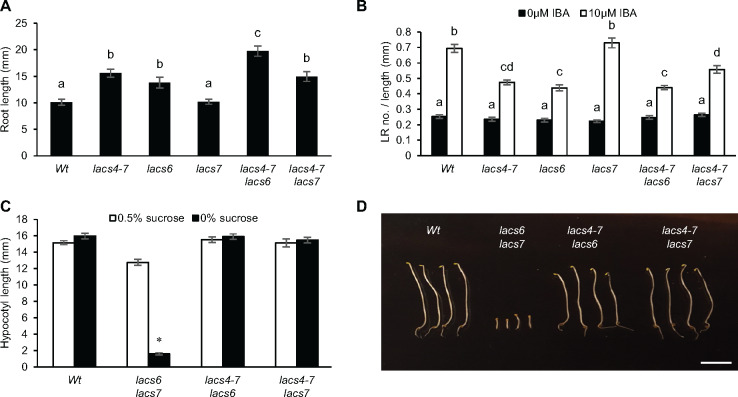
LACS4 and LACS6 have overlapping activity in IBA metabolism but not fatty acid β-oxidation. (**A**) Primary root elongation of 7-d-old seedlings on 10 *µ*M IBA. Statistical significance was determined by one-way ANOVA with *post hoc* Tukey HSD test (± se, *n* ≥ 12, *P* < 0.05). Common letters indicate no significant difference. (**B**) Lateral root density of 8-d-old seedlings was quantified by dividing the number of lateral roots by the primary root length. Statistical significance determined by two-way ANOVA with *post hoc* Tukey HSD (± se, *n* ≥ 14, *P* < 0.05). Common letters indicate no significant difference. (**C**) Hypocotyl elongation of seedlings grown with and without sucrose for 1 d in light and 5 d in darkness. Statistical significance determined by two-tailed *t* test between sucrose treatments (± se, *n* ≥ 17, *P < 0.001). (**D**) Representative hypocotyls of seedlings grown for 1 d in light and 5 d in darkness without sucrose. Scale bar = 5mm.

Nine *LACS* genes are encoded in the Arabidopsis genome (Shockey et al., 2002). As several LACS proteins have overlapping roles (Jessen et al., 2011, 2015; Zhao et al., 2019), we examined all *lacs* mutants for IBA resistance. Available T-DNA lines were screened for root elongation on IBA. We found *lacs6* is the only other *lacs* mutant resistant to IBA in primary root elongation (Supplemental Figure S2). The finding that *lacs6*, but not *lacs7*, is resistant to IBA in primary root elongation (Figure 3A) was surprising as LACS6 and LACS7 are closely related, both localized to the peroxisome, and have overlapping activity in the first step of fatty acid β-oxidation (Shockey et al., 2002; Fulda, 2004). *lacs6* is comparable to *lacs4* in that it also has slightly reduced lateral root density when treated with IBA (Figure 3B).

To further explore the relationships between LACS4, LACS6, and LACS7, double mutants of *lacs4* with *lacs6* or *lacs7* were generated. *lacs4-7 lacs6*, but not *lacs4-7 lacs7*, displayed enhanced resistance to IBA in primary root elongation when compared with single mutants (Figure 3A). This enhancement was not seen in the lateral root phenotype; *lacs4-7 lacs6* lateral root density was not statistically different from the single mutants (Figure 3B). This evidence supports that *lacs4* and *lacs6* are not defective in lateral root initiation and that their IBA-defective responses are predominately seen in the primary root. The finding that *lacs6* is resistant to IBA and enhances the IBA resistance in primary root elongation of *lacs4* suggests that LACS4 and LACS6 may work in combination in IBA to IAA conversion. These experiments also demonstrate that LACS7 does not have a role in IBA to IAA conversion.

### *lacs4* is not sucrose dependent, even in combination with *lacs6* or *lacs7*

Redundancy is common with β-oxidation enzymes (Hu et al., 2012). Single mutants may not have sucrose-dependent phenotypes even when a biochemical defect is present. For example, *lacs6* and *lacs7* appear wild-type but growth is severely compromised in a *lacs6 lacs7* double mutant (Fulda, 2004). *lacs4* did not display sucrose-dependent phenotypes (Figure 1F). The overlapping role of LACS4 and LACS6 in IBA responses led us to analyze if *lacs4* has a sucrose-dependent phenotype in higher order mutants. *lacs4-7 lacs6* and *lacs4-7 lacs7* were evaluated for sucrose dependence. Neither double mutant was defective in hypocotyl elongation on media lacking sucrose, indicating *lacs4* does not display the sucrose-dependent phenotype consistent with fatty acid β-oxidation defects (Figure 3, C and D). We conclude that fatty acid β-oxidation is not detectably compromised when *LACS4* is mutated.

### *lacs4* and *lacs6* responses are specific to auxins that are β-oxidized

**Figure 4:**
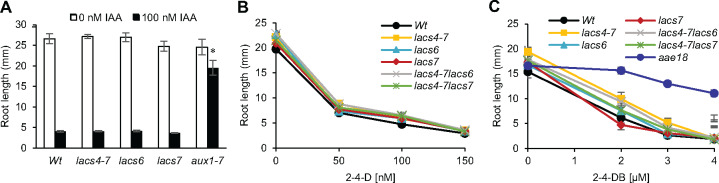
LACS4 and LACS6 are specific to auxins that must be β-oxidized. (**A**) Primary root elongation of 7-d-old seedlings grown with and without IAA. Statistical significance determined by two-tailed *t* test to *Wt* of the same treatment (± se, *n* ≥ 12, *aux1-7 n* = 8, *P < 0.001). (**B**) Primary root elongation of 7-d-old seedlings grown on increasing concentrations of 2-4-D (± se, *n* ≥ 16). (**C**) Primary root elongation of 7-d-old seedlings grown on increasing concentrations of 2-4-DB (± se, *n* ≥ 10).

Our evidence demonstrates that *lacs4* and *lacs6* are resistant to IBA. We previously showed that *lacs4* responses were specific to IBA; to explore if *lacs6* responses are specific to IBA, primary root elongation was tested on IAA and found to be inhibited similarly to wild-type (Figure 4A). In addition, we tested these *lacs* mutants on the synthetic auxin 2,4-dichlorophenoxyacetic acid (2-4-D). *lacs4*, *lacs6*, *lacs4-7 lacs6*, and *lacs4-7 lacs7* are sensitive to 2-4-D-based inhibition of primary root elongation to the same extent as wild-type (Figure 4B). This indicates that neither *lacs4* nor *lacs6* are disrupting generalized auxin responses.

*lacs4* and *lacs6* were also tested on (2,4-dichlorophenoxy)butyric acid (2-4-DB), a synthetic auxin that has a side chain that is two carbons longer than 2-4-D and may be β-oxidized in a similar mechanism to that of IBA (Wain and Wightman, 1954). We used the peroxisomal acyl-activating enzyme, *aae18*, as a control as it is specific for activating 2-4-DB for β-oxidation and remains sensitive to IBA (Wiszniewski et al., 2009). *lacs4* is resistant to 2-4-DB only at low concentrations and not to the same extent as *aae18* (Figure 4C). *lacs6* remained sensitive to 2-4-DB (Figure 4C), consistent with previously published results (Fulda, 2004). Resistance to 2-4-DB was not enhanced in the *lacs4-7 lacs6* double mutant, supporting that *lacs6* is sensitive to this auxin. The differential effect of LACS4 and LACS6 on 2-4-DB may indicate that LACS4 preferentially participates in auxin β-oxidation compared to LACS6 or that LACS4 may be capable of accepting a broader range of substrates than LACS6.

### LACS4 localization

All proteins identified in IBA to IAA conversion are in the peroxisome matrix or have roles in general peroxisome function (Hu et al., 2012). However, a previous study (Jessen et al., 2015) reported LACS4 localizes to the endoplasmic reticulum (ER). Based on the phenotypes described above and the potential connection of LACS4 with IBA activation, we hypothesized LACS4 also would associate with peroxisomes.

**Figure 5:**
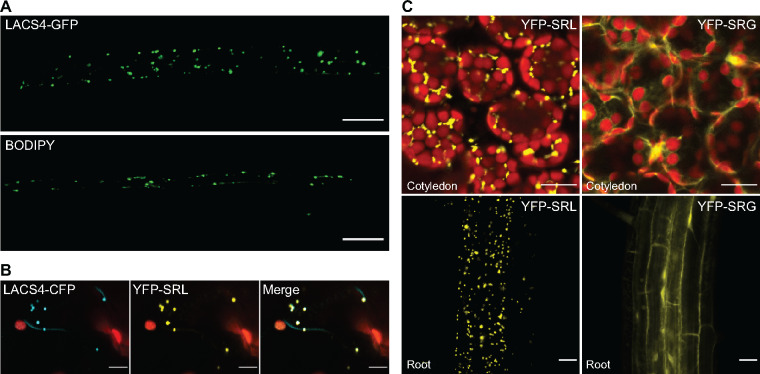
LACS4 localizes to puncta reminiscent of peroxisomes. (**A**) Visualization of LACS4-GFP and BODIPY signals in peroxisomes of 7-d-old Arabidopsis. Scale bar = 20 *µ*m. (**B**) Localization of transiently expressed LACS4-CFP and YFP-SRL in *Nicotiana benthamiana*. Scale bar = 10 *µ*m. (**C**) Representative confocal images of YFP-SRL and YFP-SRG signals in cotyledons and roots of 7-d-old transgenic Arabidopsis. Scale bar = 20 *µ*m.

A full-length *LACS4* cDNA was used to generate a C-terminal GFP fusion with a flexible linker between LACS4 and the fluorescent tag. This construct was transformed into wild-type and *lacs4-8* plants*.* This construct rescued the IBA-resistant phenotype of *lacs4-8*, demonstrating that the construct is functional (Figure 2C). LACS4-GFP could be viewed as distinct puncta. These puncta looked comparable in size and number to peroxisomes stained with the peroxisome-specific dye BODIPY (Landrum et al., 2010) in wild-type seedlings (Figure 5A).

To determine if these puncta were peroxisomes, *Nicotiana benthamiana* was co-infiltrated with *Agrobacterium tumefaciens* containing a 35S:*LACS4-CFP,* a cyan fluorescent protein attached to the *LACS4* coding sequence with a flexible linker, and UBQ10:*YFP-*SRL, a yellow fluorescent protein ending with a canonical PTS1. Transient expression of LACS4-CFP could be seen as discrete puncta that colocalized with the peroxisome marker (Figure 5B). This result supports our hypothesis that LACS4 can associate with peroxisomes.

Proteins internalized into the peroxisome typically contain a PTS. LACS4 does not have a canonical PTS1 or PTS2. However, the PTS1 C-terminal tripeptide (typically [S] [RK] [LM]), can accept degeneracy at these residues (Chowdhary et al., 2012). The LACS4 C-terminal amino acids are SRG, one amino acid different from a canonical PTS1. To test if the C-terminal tripeptide of LACS4 functions as a noncanical PTS, a UBQ10:*YFP* construct was mutated to end either with SRG or SRL terminal amino acids. YFP-SRL localized as discrete puncta (Figure 5C), demonstrating peroxisome localization. In contrast, YFP-SRG was seen as a diffuse fluorescent signal (Figure 5C). This experiment demonstrates that if LACS4 is entering into the peroxisome, it is doing so by a different mechanism than a C-terminal tripeptide PTS or that additional upstream residues are required beyond the consensus amino acids. Alternatively, LACS4 may associate with the peroxisome without being internalized.

### LACS4 directly acts on IBA

**Figure 6:**
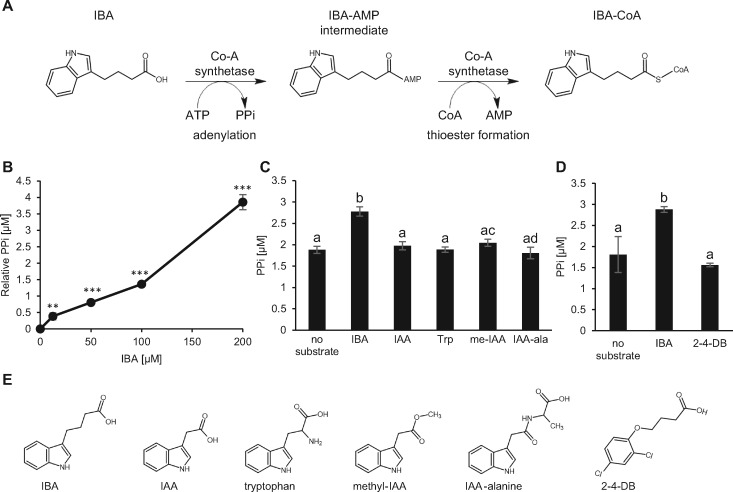
LACS4 has activity on IBA substrate *in vitro*. (**A**) CoA synthetase reaction on IBA depicting intermediates and byproducts. (**B**) Recombinant LACS4 was incubated with increasing concentrations of IBA dissolved in water for 10 min *in vitro*. Production of PPi was measured and represented relative to *µ*M PPi detected without substrate. Statistical significance was determined by a two-tailed *t* test (±SE, *n* = 6, **P<0.01, ***P<0.001). (**C**) Recombinant LACS4 was incubated with 100 μM of IBA, IAA, tryptophan (Trp), methyl-IAA (me-IAA), and IAA-alanine (IAA-Ala) for 10 min *in vitro* before PPi was measured. Statistical significance was determined by one-way ANOVA with *post hoc* Tukey HSD test (± se, *n* = 6, *P* < 0.01). Common letters indicate no significant difference. (**D**) Recombinant LACS4 was incubated with 100 μM of IBA, 2-4-DB, or no substrate for 10 min in vitro before PPi was measured. Statistical significance was determined by one-way ANOVA with *post hoc* Tukey HSD test (± se, *n* = 6, *P* < 0.05). Common letters indicate no significant difference. (**E**) Chemical structures of IBA, IAA, tryptophan, methyl-IAA, IAA-alanine, and 2-4DB.

LACS4 CoA synthetase activity on fatty acids has been demonstrated (Shockey et al., 2002). The IBA-resistant phenotype of *lacs4* suggests that LACS4 also is involved in IBA metabolism. To determine if the mechanism of IBA resistance of *lacs4* is due to a wild-type role for CoA synthetase activity on IBA, *in vitro* enzyme activity assays were performed with recombinant LACS4. A CoA synthesis reaction consumes ATP to yield pyrophosphate (PPi) and AMP (Figure 6A). We examined enzyme activity by measuring the production of PPi with a fluorometric assay. After 10 min of incubation with IBA, LACS4 produced significantly more PPi than when incubated with no substrate, and PPi production increased with increasing concentrations of IBA (Figure 6, B and C).

To evaluate substrate specificity, LACS4 was tested on IAA, IAA-alanine, methyl-IAA, and tryptophan, an auxin precursor. These substrates were chosen because each are endogenous, relevant to auxin metabolism, and structurally similar to IBA. Each possess an indole ring identical to that of IBA but have side chains of varying lengths and modifications (Figure 6E). Chain length is of interest because LACS4 has activity on fatty acids ranging in length from 14 to 18 carbons (Shockey et al., 2002). Methyl-IAA was included because it has a side chain as the same length of IBA, but does not have a terminal carboxyl group to participate in adenylation. Despite significant LACS4 activity on IBA, activity was not detected for IAA, IAA-alanine, methyl-IAA, or tryptophan (Figure 6C). This result demonstrates that LACS4 has specific activity on IBA, but not other structurally similar auxins or carboxylic acids. This biochemical data are consistent with *lacs4* being resistant specifically to the effects of IBA.

We expanded our *in vitro* analysis of LACS4 substrates to include 2-4-DB. 2-4-DB is a synthetic auxin and not endogenous to plants. *lacs4* is weakly resistant to 2-4-DB at low levels. However, LACS4 did not have activity on 2-4-DB (Figure 6D), suggesting that it is not a bona fide substrate.

Evidence that LACS4 has increasing activity with increasing IBA substrate and exhibits specificity for IBA over related auxins strongly supports that LACS4 directly acts on IBA and can charge IBA with the necessary CoA before entering the β-oxidation pathway.

### *lacs4* has reduced auxin signaling in root tip

**Figure 7:**
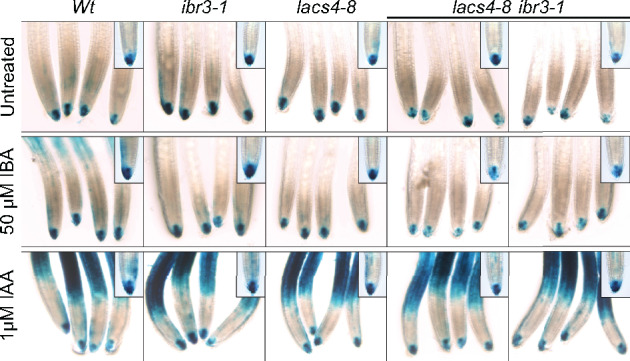
*lacs4* and *lacs4 ibr3* display reduced IBA-responsive DR5 activation. Staining of DR5:GUS in root tips of 5-d-old seedlings. Plants were incubated in liquid PN with or without hormone gently rocking for 2 h. Plants were stained for GUS for 3 h at 37°C and washed with 50, 75, and 95% EtOH before imaging. Groups were imaged with a 20× objective, inset is 40× objective.

IBA conversion to IAA is an important source of auxin for root development (Strader and Bartel, 2011; De Rybel et al., 2012; Xuan et al., 2015) and contributes to establishing and maintaining the total auxin pool (Spiess et al., 2014). To examine if LACS4 can influence overall auxin signaling, *lacs4-8* and *lacs4-8 ibr3-1* were crossed to DR5:GUS, a reporter line that contains the synthetic auxin-responsive reporter DR5 driving expression of the β-glucoronidase (GUS) enzyme (Ulmasov et al., 1997). Seedlings were evaluated for relative strength of the reporter in root caps, the hypothesized location of IBA conversion into IAA (De Rybel et al., 2012; Xuan et al., 2015). In media without hormone, staining in wild-type, *ibr3*, and *lacs4-8* was comparable. DR5:GUS induction was slightly reduced in *lacs4-8 ibr3-1*. We hypothesize this to be due to reduced metabolism of endogenous IBA into IAA in the root cap. Upon treatment with IBA, wild-type plants showed increased and expanded induction of the DR5:GUS construct. However, *lacs4-8* and *lacs4-8 ibr3-1* were not induced to the same levels as wild-type (Figure 7), suggestive of reduced auxin signaling (Ulmasov et al., 1997). Staining was similar in all genotypes incubated with IAA (Figure 7), demonstrating that the seedlings still retain the ability to sense and respond to IAA; only the IAA generated from the breakdown of IBA is compromised. The reduced GUS induction in *lacs4* supports a hypothesis that LACS4 is functionally contributing to the total auxin pool available in the root cap and is doing so through the mechanism of IBA to IAA conversion.

## Discussion

IBA metabolism to IAA parallels fatty acid β-oxidation; enzymes with similar activities likely will be required for the analogous steps. It is hypothesized that IBA is charged with CoA by a CoA synthetase before entering the pathway. Our identification of LACS4 in an unbiased screen for IBA resistance was intriguing, as a CoA synthetase functioning on IBA had not yet been reported. Separation of the new allele from the previously characterized *ibr3* IBA-response mutant background (Zolman et al., 2000; Zolman et al., 2007) revealed that *lacs4* has a notable IBA-resistance profile on its own. Mutations in *LACS4* make Arabidopsis resistant to the inhibition of IBA in primary root elongation and dark-grown hypocotyl elongation (Figure 1, A and D), consistent with previously characterized IBA-response mutants (Zolman et al., 2000; Zolman et al., 2007, 2008; Strader et al., 2011).

LACS4 CoA synthetase activity is specific to IBA *in vitro.* LACS4 was not active against other auxin substrates and *lacs4* remains sensitive to IAA in primary root elongation (Figures 4, 6). Our data support a model in which LACS4 catalyzes CoA addition onto IBA, the first step in IBA to IAA conversion, and is required for normal contribution of IBA-derived IAA in the root.

### LACS4 function

LACSs in all organisms, from prokaryotes to humans, primarily activate fatty acids with CoA making LACS enzymes critical for utilization of fatty acids (Groot et al., 1976; Watkins, 1997). The Arabidopsis genome contains nine *LACS* genes with some overlapping activity, but which vary in substrate specificities, localization, and expression (Shockey et al., 2002; Zhao et al., 2019). Teasing apart the role of LACSs has been difficult as most single mutants have no obvious lipid phenotypes. Higher-order mutants have revealed specific roles of some Arabidopsis LACS proteins.

LACS4 has overlapping activity with LACS1, LACS2, LACS8, and LACS9 with roles in a diverse array of lipid biosynthesis. *lacs4* appears wild-type in vegetative growth, fertility, and seed oil content (Jessen et al., 2011, 2015; Zhao et al., 2019), but phenotypes began to emerge upon combining *lacs4* with other mutants. LACS4 and LACS9 act in glycerolipid synthesis and work together to transfer lipids from the ER to the plastid. *lacs4 lacs9* had reduced stature, reduced seed weight, altered seed oil content, and impaired seed development (Jessen et al., 2015; Zhao et al., 2019). LACS8 also may have overlapping activity as a *lacs4 lacs8 lacs9* triple mutant is embryo lethal (Jessen et al., 2015; Zhao et al., 2019). LACS4 and LACS1 together are required for synthesis of pollen coat lipids, as *lacs1 lacs4* shows male sterility (Jessen et al., 2011). *lacs1 lacs2 lacs4* has altered seed oil content and dramatically reduced cuticle waxes (Zhao et al., 2019). These studies link LACS4 activity to vegetative growth, seed oil accumulation, and fertility, highlighting that LACS4 has a wide impact on growth and fecundity.

Our results implicating LACS4 activity in IBA metabolism expands the roles for LACS4 to span lipid and hormone metabolism. Our work reveals LACS4 also influences early seedling development, particularly root systems, through IBA to IAA conversion and contributes to the overall pool of free IAA, as indicated by reduced induction of DR5:GUS in *lacs4-8 ibr3-1* in the root cap (Figure 7).

Mirroring the overlapping roles of LACS family enzymes described above, our study also implicated LACS6 as another CoA synthetase that can act on IBA. LACS6 is localized in the peroxisome matrix (Fulda, 2004; Jessen et al., 2015), consistent with our initial hypothesis that the acyl-CoA synthetase acting on IBA would also be localized where all other IBA metabolizing enzymes are located (Hu et al., 2012). *lacs6* is resistant to IBA in primary root elongation (Figure 3) at levels comparable to *lacs4*, supporting the hypothesis that LACS6 acts directly on IBA. Alternatively, *lacs6* could indirectly disrupt IBA metabolism. Disruption to *LACS6* could be indirectly influencing IBA metabolism via a mechanism such as CoA limitation, slowed peroxisome metabolism due to accumulation of fatty acid intermediates, or accepting exogenous IBA as substrate because it is in excess. Such a model might suggest *lacs7* also would display IBA resistance, as LACS6 and LACS7 are both highly expressed in the same tissues and have overlapping activity on fatty acid substrates (Shockey et al., 2002). However, whereas *lacs6* is resistant to IBA, *lacs7* has wild-type responses to IBA in all phenotypes tested and no additional enhancement was noted in the *lacs4 lacs7* double mutant in primary root elongation or lateral root initiation assays. Future work characterizing the substrate profile of LACS6 will be necessary to establish a direct or indirect role of LACS6 in IBA metabolism.

*lacs4* and *lacs6* single mutants have IBA-resistant phenotypes (Figure 3), whereas defects in fatty acid β-oxidation, seed oil accumulation, and glycolipid synthesis are only seen in higher-order mutants with other *lacs* mutants (Figure 3 and Shockey et al., 2002; Fulda, 2004; Jessen et al., 2015).

Together these findings illustrate that activity of LACS4 and LACS6 on IBA is unique, although expanded studies of this enzyme family will reveal additional details of enzyme activity and pathway interactions. For instance, *lacs8-2* shows a slight hypersensitivity to IBA (Supplemental Figure S2); *lacs8* is of interest as other studies report overlapping activity of LACS4 and LACS8 (Jessen et al., 2015; Zhao et al., 2019). *lacs3* also has a slight hypersensitivity to IBA but has reduced primary root length without hormone treatment, potentially indicating a general growth defect instead of an IBA-specific effect. Other LACS enzymes have not been thoroughly investigated yet, but future experiments will reveal their roles on fatty acids and potentially other related substrates.

### Additional interactions may be required for full IBA responses in Arabidopsis

*lacs4* develops lateral roots in response to IBA stimulation, although not to the same extent as wild type (Figures 1, 3); this differential pattern of resistance across developmental stages is rare in IBA-response mutants. For instance, *acx3* also develops lateral roots in the presence of IBA but is resistant in primary root elongation (Eastmond et al., 2000; Adham et al., 2005). The distinct primary and lateral root IBA-responses of *acx3* is attributed to its expression pattern. *ACX3* expression in roots is seen in the root tip and tips of elongated lateral roots but not lateral root primordia, even upon IBA treatment (Eastmond et al., 2000; Adham et al., 2005).

The mechanism for LACS4 differential responses remains an open question. *LACS4* and *LACS6* are highly expressed in root tissue compared to other LACSs (Shockey et al., 2002) and analysis of a LACS4 promoter:GUS construct demonstrated that *LACS4* expression is detected in the root tip but is absent from the elongation zone (Zhao et al., 2019). Analysis of *LACS4* expression in lateral root primordia is required to determine if the different IBA response phenotypes of *lacs4* can be attributed to cell-specific expression or activity.

Interestingly, *lacs6* has been previously characterized and shows sensitivity to the synthetic IBA analog 2,4-DB (Fulda, 2004), similar to our results (Figure 4C). AAE18 is an acyl-activating enzyme found in peroxisomes with predicted overlapping activity. Notably, *aae18* mutants are sensitive to IBA, but resistant to 2,4-DB (Figure 3C; Wiszniewski et al., 2009). This unique pattern of responses represents another area for future investigation, as connections and overlap between LACS4, LACS6, and AAE18 could be revealing for different tissues or conditions as suggested by their unique but overlapping phenotypes.

### Unique aspects of *lacs4-8* mutation

Our *lacs4* allele has a weak missense mutation of an alanine to valine. Residue 467 is not located in a known substrate or cofactor binding pocket or active site (Figure 2). Our structural analysis and functional prediction using Phyre2 (Kelley et al., 2015) suggest this residue is not in a functionally important domain or region of secondary structure. Our genetic evidence that a substitution at residue 467 disrupts function unveils questions about which regions in LACS4 are important for function and if they are equally important for lipid and IBA metabolism.

We also completed mutational analysis using the SuSPect method (Yates et al. 2014). Substitution to any amino acid at residue 467 is predicted to have near neutral effects. However, despite this prediction, there is no amino acid degeneracy at this residue; this residue is a completely conserved alanine in all *A. thaliana* accessions and a proline in all other Arabidopsis LACS proteins and LACS4 orthologs in other plant species, including closely related Arabidopsis species (Figure 2). Such conservation suggests an evolutionary pressure to maintain the amino acids at this site. It is striking that *LACS4* of *A. thaliana* encodes a divergent alanine at this site. The PAM250 matrix (Schwarz and Dayhoff, 1979) predicts that proline is most often and equally likely to be substituted for alanine or serine. Although the recent divergence of *A. thaliana* at this amino acid is surprising, a substitution for alanine may be favorable and necessary for function. This biological evidence of strong conservation increases our confidence that this residue is sensitive to perturbations, despite bioinformatics predictive analysis.

Our genetic evidence supports that residue 467 is important for protein function, folding, or stability, given that a point mutation disrupts plant physiology to the same extent as null alleles (Figure 2E). Continued analysis of the LACS4 domain structure generally and this region specifically is required to determine if this site is required for binding to a substrate, interacting protein, or required to give the protein a precise structure.

### LACS4 localization remains an open question

In our analysis of localization, LACS4-GFP could be viewed as distinct puncta in Arabidopsis (Figure 5). These puncta looked comparable to BODIPY-stained peroxisomes. The punctate localization was also seen when LACS4-CFP was transiently expressed in *N. benthamiana* and colocalized with YFP:SRL-labeled peroxisomes. This evidence that LACS4 associates with peroxisomes is consistent with localization of other IBA to IAA enzymatic components (Hu et al., 2012). Subcellular localization prediction tools yield disparate predictions. WoLF-PSORT (Horton et al., 2007) and Suba4 (Hooper et al., 2017) most often predict cytosol localization followed by peroxisome localization, but also predict localization to all other cellular compartments.

If LACS4 is localized to the peroxisome matrix, it is unclear how import occurs as LACS4 does not possess a canonical PTS1 or PTS2. The LACS4 C-terminal amino acids SRG are not a sufficient PTS for entry into the peroxisome (Figure 5), although additional upstream amino acids not tested could facilitate import (Brocard and Hartig, 2006; Chowdhary et al., 2012). Some peroxisomal proteins without a PTS piggyback onto PTS containing proteins (Thoms, 2015) and protein oligomers can be imported even when subunits lack a PTS (Brown and Baker, 2008). This piggybacking mechanism has been reported for protein phosphatase 2A in Arabidopsis (Kataya et al., 2015) and even proteins that are not part of a complex, such as Nicotinamidase I which is co-imported into the peroxisome with glycerol-3-phosphate dehydrogenase 1 in *Saccharomyces cerevisiae* (Saryi et al., 2017).

LACS4 previously has been described as ER localized in Arabidopsis (Jessen et al., 2015) and *Brassica napus* (Tan et al., 2014). However, the mammalian ortholog, namely *ACS4*, localizes to the mitochondria, ER, and peroxisomes (Lewin et al., 2001, 2002; Milger et al., 2006; Grevengoed et al., 2014). This finding opens the question of LACS4 localization and the possibility of dual localization. A detailed study of LACS4 throughout development and under changing conditions, as well as identification of an interacting protein that could serve as a vehicle into the peroxisome, is necessary to elucidate the localization of LACS4 and understand the implications of potential dual localization over space and time.

### Potential mechanisms of LACS4 activation of IBA

**Figure 8:**
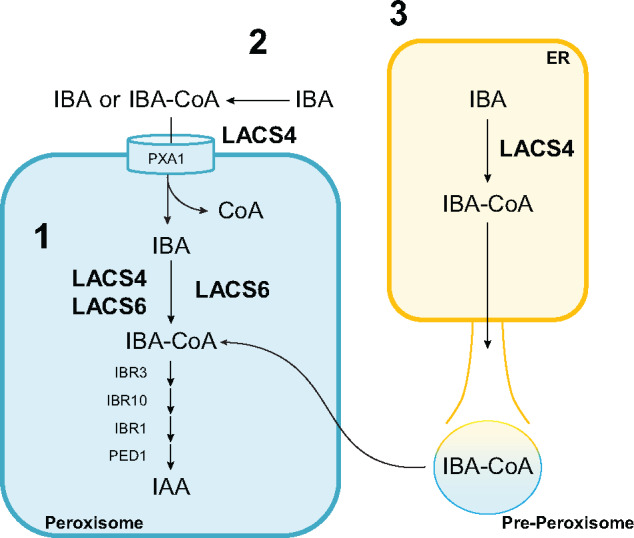
Three possible models of LACS4 activation of IBA, which affects IBA metabolism to IAA in the peroxisome. (1) LACS4 and LACS6 are both located inside the peroxisome where together they charge IBA with CoA. LACS4 may localize solely to the peroxisome or also localize to the ER and/or cytoplasm. (2) LACS4 may exist in the cytoplasm and loosely associated with the peroxisome without being internalized by the organelle to generate a cytoplasmic pool of IBA-CoA. IBA-CoA is imported into the peroxisome where LACS6 can reattach the CoA onto IBA. (3) ER-localized LACS4 may generate a pool of IBA-CoA that is actively imported into the peroxisome or is contained within pre-peroxisome structures that become mature peroxisomes. CoA cleaved off during import is reattached by the peroxisomally contained LACS6.

Our genetic and biochemical data support that LACS4 charges IBA with CoA. Genetic evidence suggests LACS6 does this as well. We hypothesize these two enzymes work together at this step via one of three possible mechanisms (Figure 8).

All enzymatic components of IBA to IAA conversion discovered to date are contained within the peroxisome. Our LACS4 localization experiments demonstrate LACS4 could be localized in the peroxisome matrix as well. This localization supports the hypothesis that LACS4 and LACS6 together charge IBA with CoA inside the peroxisome.

Alternatively, LACS4 may reside outside of the peroxisome, associated with the outer membrane. LACS4 generates a cytosolic pool of IBA-CoA, which is imported into the peroxisome. Active import by PXA1 is coupled with CoA hydrolysis (Nyathi et al., 2010; Lousa et al., 2013) and in this model, IBA could be reactivated by peroxisomal LACS6 in the matrix.

However, previous work also has reported LACS4 localization in the ER (Jessen et al., 2015). LACS4 may generate a pool of IBA-CoA in the ER for import into the peroxisome, which could be reactivated by LACS6.

Finally, there could be two pools of LACS4 with distinct functions. One pool may be ER localized and function primarily in fatty acid metabolism, whereas a peroxisomal pool may be primarily associated with hormone metabolism. Such a model is consistent with our mutant analysis suggesting loss of *LACS4* affects IBA responses, but does not affect peroxisomal lipid metabolism during early seedling development.

Each of these proposed mechanisms have a novel aspect that is intriguing. If LACS4 is peroxisomal, then it is unclear how it is imported without a canonical PTS. LACS4 could enter via the piggyback mechanism, an extended signal sequence, or a noncanonical PTS. If LACS4 is solely localized to the ER, it is the first enzyme with a role in IBA metabolism not linked to the peroxisome. However, peroxisomes have a close relationship with the ER. Peroxisomes bud off the ER membrane to become independent entities, as described in a model of de novo biogenesis, or the ER may serve as a source of membranes and membrane proteins, as described in a semi-autonomous model (Mullen and Trelease, 2006; Hu et al., 2012; Agrawal and Subramani, 2016). IBA-CoA generated from LACS4 in the ER may be moved into the peroxisome via these membrane fusion models rather than active import. If LACS4 is only localized to the ER, this furthers the growing evidence that implicates the ER in auxin metabolism. IBA can be formed via acetylation of IAA and is associated with the ER membrane in maize (*Zea mays*; Ludwig-Müller and Epstein, 1992; Ludwig-muller and Hilgenberg, 1995). Auxin-conjugate hydrolases reside here, IAA actively is transported into the ER, and the ER may regulate the amount of auxin transported to the nucleus for signaling (Bartel and Fink, 1995; Davies et al., 1999; Friml and Jones, 2010; Middleton et al., 2018).

Finally, LACS4 may exist in both the ER and the peroxisome. Multiple pools of LACS4 would accommodate the pleiotropic activity of LACS4 in lipid and IBA metabolism. Localization to multiple organelles is seen in the mammalian isoform ACS4, which is localized to the ER, peroxisome, and mitochondrial membrane (Grevengoed et al., 2014). In mammals, superoxide dismutase and lactate dehydrogenase are localized primarily to the cytosol with a smaller peroxisomal pool that enter via piggybacking mechanisms (Wanders and Waterham, 2006; Islinger et al., 2009). The import of these proteins into the peroxisome is dependent on the amount of available carrier protein, offering an explanation for the comparatively reduced amount of protein in the peroxisome, which is often masked in in vitro studies (Thoms, 2015). Dual localization of ACS4 and the ability of superoxide dismutase and lactate dehydrogenase to enter the peroxisome without PTSs while maintaining primary localization elsewhere substantiates the possibility that LACS4 could have also dual localization in Arabidopsis and suggests a rationale for why LACS4 does not have a PTS, why peroxisomal localization has not been previously detected, and why a significant role in fatty acid β-oxidation is not observed.

## Summary

The roles of IBA-derived IAA, and auxin storage forms more broadly, have been investigated in detail but continue to be studied to determine the activity and relative importance of each throughout plant growth, development, and responses to changing environmental conditions. Identification of LACS4 as an activator of IBA provides a more complete view of the IBA metabolic pathway, facilitating additional studies to increase our understanding of alternative auxin input pathways during plant development. In addition, this work gives new light to the role of LACS4 within a cell and the importance of LACS4 in plant development. LACS4 stands at the crossroads of hormone and lipid metabolism, two molecular pathways required to balance growth, development, and responses to the environment throughout the lifespan of a plant.

## Materials and methods

### Plant materials

All plants are *A. thaliana* of the Columbia (Col-0) background. *ibr3-1* has a point mutation generated by EMS (Zolman et al., 2007). *ibr3-4* (SALK_004657), *lacs1-2* (SALK_138782), *lacs2-3* (GABI_368C02), *lacs3-1* (SALK_ 027707), *lacs4-1* (SALK_101543), *lacs4-7* (SALK_120357), *lacs6* (SALK_069510c), and *lacs7* (SALK_146444), *lacs8-2* (SALK_105118), and *lacs9-4* (SALK_124615) are T-DNA lines obtained from the Arabidopsis Biological Resource Center. All mutants were genotyped to confirm homozygous mutations (Supplementary Tables S2 and S3).

*lacs4-1*, *lacs4-7*, *lacs6*, and *lacs7* were backcrossed to wild type, reisolated by genotyping and confirmed homozygous. All double mutants were obtained from directed outcrossings.

### Mutagenesis and mutant isolation

*ibr3-1* seeds were soaked in 0.24% EMS (v/v) for 18 h in the dark, washed extensively, and transferred to soil. M_2_ seedlings were grown on 25 *µ*M IBA and screened for individuals with elongated roots compared to the *ibr3-1* background. M_3_ progeny were retested to validate the mutant phenotype.

*Z377 ibr3-1* was backcrossed to *ibr3-1* to generate lines for sequencing. *Z377* was isolated from segregating generations of the *Z377 ibr3-1* backcross to wild type by screening for seedlings with an intermediate IBA-resistant root phenotype that genotyped wild-type for *ibr3-1*. *Z377 ibr3-1* and *Z377* were backcrossed to wild-type twice to generate lines for phenotypic analysis.

### Growth conditions for phenotypic assays

Seeds were sterilized with 30% bleach (v/v) and 0.1% triton (v/v), rinsed, suspended in 0.1% agar (w/v), and imbibed at 4°C for 3–5 d. Seeds were plated on plant nutrient media (PN; Haughn and Somerville, 1986) supplemented as indicated. Plates were incubated at 22°C under continuous yellow light for the specified number of days.

Primary root length was measured after 7 d of growth on indicated media. Lateral root density was determined by growing seeds on PN for 4 d, transferring seedlings to PN or PN + 5 *µ*M IBA plates and growing for an additional 4 d. Lateral roots were counted with a Leica Zoom 2000.

Sucrose dependence and effects of auxins on hypocotyl length were performed by growing seeds on indicated media for 1 d. Plates were then incubated in darkness for an additional 5 d before hypocotyls were measured.

### Whole-genome sequencing and mutant genotyping

Ten independently isolated *Z377 ibr3-1* lines from a backcross to *ibr3-1* were selected and pooled for sequencing in parallel with wild type and *ibr3-1*. DNA was extracted from approximately 2,000 seedlings grown on filter paper for 10 d. Sequencing was performed at the Genome Technology Access Center at Washington University in St Louis on an Illumina-HiSeq2000. Gene candidates were first narrowed down by identifying homozygous single nucleotide polymorphisms consistent with EMS mutagenesis in coding regions. Mutations common between *Z377 ibr3-1* and wild type or *ibr3-1* were eliminated, leaving mutations unique to *Z377 ibr3-1.* dCAPS primers (Neff et al., 2002) were designed to genotype the point mutation in *lacs4-8* (Supplemental Table 2).

### Generation of constructs

*LACS4* cDNA was amplified with LACS4Topo-F and LACS4Topo-R from cDNA synthesized from wild-type RNA. *LACS4* was cloned into the Gateway entry vector pcr8/Topo. The stop codon of LACS4 was replaced with a 6-amino-acid, glycine-serine flexible linker with the Q5 Site Directed Mutagenesis Kit (NEB) using the primers LACS4-cterm linker F and LACS4 c-term linker R. This *LACS4* construct was moved into plant gateway expression vectors pEarlyGate102 and pEarlyGate103 (Earley et al., 2006) with LR clonase (Thermo Fisher).

For recombinant expression, *LACS4* cDNA was amplified with primers LACS4S and LACS4B, digested with Sal1-HF and BamHI-HF, and ligated into the pZA31 expression vector which adds an N-terminal HN tag and controls expression through the TetR system (Lutz and Bujard, 1997). pZA31:*LACS4* was transformed into *Escherichia coli* DH5ɑZ1, a strain modified to constitutively express *TetR* from the chromosome for bacterial expression (Lutz and Bujard, 1997).

UBQ10:*YFP*-GW (Michniewicz et al., 2015) was mutated by site-directed mutagenesis to end with SRL and SRG C-terminal tripeptides with the primers YFP-SRG F, YFP-SRG R, YFP-SRL F, and YFP-SRL R using the Quikchange XL site-directed mutagenesis kit (Agilent Technologies).

Constructs were confirmed by sequencing. Primer sequences are listed in Supplementary Table S3.

### Plant transformation

Wild-type and *Z377* were transformed with the pEarlyGate103:*LACS4* fluorescent fusion protein construct. UBQ10:YFP-SRL and UBQ10:YFP-SRG were independently transformed into wild-type. Arabidopsis transformation was done via the floral-dip method with *Agrobacterium tumefaciens* (Clough and Bent, 1998). Transformants were selected by growing seedlings on 10 *µ*g mL^−1^ Basta for 14 d. Homozygous lines were identified by screening T_3_s on Basta.

### Transient LACS4 expression

LACS4 localization was observed through transient expression in *N. benthamiana* (Sparkes et al., 2006). pEarlyGate102:*LACS4* and UBQ10:*YFP*-SRL in *A. tumefaciens* were grown overnight at 30°C. Cultures were incubated in infiltration media (10 mM MgCl_2_, 10 mM MES, and 200 µM acetosyringone) and gently rocked at room temperature for 4 h. The cells were resuspended in fresh infiltration media to an OD_600_ of 1. Cultures of each construct were mixed at a ratio of 1:1 and infiltrated into leaves of young *N. benthamiana* plants using a 1-mL needleless syringe. Leaves were imaged 48 h after infiltration.

### Confocal microscopy

Confocal images were obtained with a Zeiss LSM 700 laser scanning confocal microscope using the 20X lens. pEarlyGate103:LACS4 was imaged in 7-d-old Arabidopsis seedlings of T_2_s showing rescued IBA-resistant phenotypes when grown on 15 µM IBA. Seven-day-old wild-type Arabidopsis seedlings were stained with 5 µM 8-(4-Nitrophenyl) BODIPY (Toronto Research Chemicals) for 10 min and excited with a 488-nm laser.

### RNA extraction and expression quantification

Seedlings were grown on filter paper under white light for 5 d. RNA was extracted by grinding seedlings in liquid nitrogen and using the IBI Total RNA Mini Kit (Plant; MidSci). cDNA was synthesized using ProtoScript II Reverse Transcriptase (NEB). *LACS4* expression was determined by RT-qPCR using Bullseye EvaGreen qPCR Mix (MidSci) on a BioRad CFX96 Real Time PCR System. *LACS4* expression was normalized to *UBIQUITIN 10* and calculated with the ΔΔCT method. Primers were designed with QuantPrime (Arvidsson et al., 2008) and are listed in Supplemental Table S3. No reverse transcriptase reactions were used as a control. Experiments were performed with both biological and technical triplicates.

### Gus staining

The DR5:GUS reporter line (Ulmasov et al., 1997) was crossed to *ibr3-1* and *lacs4-8 ibr3-1*. Five-day-old seedlings were then incubated in liquid PN with no hormone, 50 *µ*M IBA, or 1 *µ*M IAA and gently rocked at room temperature for 2 h. GUS was visualized by incubating seedlings in 0.5 mg mL^−1^ X-Gluc for 3 h at 37°C, followed by ethanol (EtOH) washes (Bartel and Fink, 1994). Plants were stored in 50% glycerol (v/v) at 4°C and imaged with Evos XL Core (Thermo Fisher) on 20× and 40× objectives.

### LACS4 purification from *Escherichia coli* DH5ɑZ1

DH5ɑZ1 containing the pZA31*:LACS4* construct was grown with 25 *µ*g mL^−1^ chloramphenicol at 37°C to an OD_600_ of 0.5. *LACS4* expression was induced with 200 *µ*g mL^−1^ anhydrotetracycline, then grown overnight at 16°C. Cells were pelleted, resuspended in 50 mM Tris pH 7.5, 300 mM NaCl, 0.1% Triton X-100 (v/v), and Pierce Protease Inhibitor Mini Tablets (Thermo Fisher). Cells were lysed by incubating with 2 mg/mL lysozyme at 4°C for 1 h followed by sonication with 10 30-s pulses. Cell debris was removed by centrifuging for 30 min at 20,000*g* at 4°C. Supernatant was then passed through a 0.22-µM filter to remove remaining debris.

The filtered supernatant was passed through an equilibrated 5-mL cobalt column on an ÄKTA prime FPLC (GE Healthcare) to capture HN-tagged LACS4. LACS4 recombinant protein was eluted in 1-mL fractions with 50 mM Tris pH 7.5, 300 mM NaCl, with increasing imidazole gradient up to 200 mM. Eluted fractions containing LACS4 were pooled and dialysed at 4°C in 10 mM Tris pH 8, 150 mM NaCl, 1 mM EDTA, and 40% glycerol (v/v). Protein concentration was determined by Bradford assay. LACS4 was aliquoted and stored at –80°C.

### LACS4 activity

LACS4 CoA synthetase activity was assayed by measuring PPi production. A 1 *µ*g of LACS4 was incubated in a 50-µL reaction of 0.5 mM CoA, 45 mM ATP, 0.1% Triton X-100 (v/v), 1 mM dithiothreitol, 100 mM Tris pH 7.5, and the indicated substrate for 10 min at 24°C. 10-mM stocks of IBA potassium salt (Chem Cruz), IAA sodium salt (Cayman Chemical Co), IAA-alanine (Milipore-Sigma), methyl-IAA (Milipore-Sigma), tryptophan, and 2-4-DB dissolved in 50% EtOH (v/v) were tested as substrates. LACS4 incubated with 5% EtOH (v/v) served as the negative control. PPi was measured with a Pyrophosphate Assay Kit (Millipore-Sigma) according to instructions by adding 50 *µ*L of reaction buffer to each CoA synthetase reaction and incubating 15 min at 24°C. PPi fluorescence was measured with *λ*_ex_=316 nm, *λ*_em_= 456 nm on a Biotek Cytation 3.

### Accession numbers

Gene sequences in this study can be found in Arabidopsis Genome Initiative database under the following accession numbers: *IBR3*, AT3G06810; *LACS4*, AT4G23850; *LACS6*, AT3G05970; *LACS7*, AT5G27600; *LACS1*, AT2G47240; *LACS2*, AT1G49430; *LACS3*, AT1G64400; *LACS8*, AT2G04350; *LACS9*, AT1G77590; *AUX1*, AT2G38120; *AAE18*, AT1G55320; *UBQ10*, AT4G05320. Sequences for long chain Co-A synthetase 4 orthologs can be accessed through Genbank under the following accession numbers: *Arabidopsis halleri*, ACC91252.1; *Brassica napus*, NP_001302548.1; *Zea mays*, XP_008652385.1, *Populus trichocarpa*, XP_024453969.1, *Amborella trichopoda*, XP_006850521.1, *Physcomitrella patens*, XP_024370732.1.

## Supplemental Data

The following materials are available in the online version of this article.

**Supplemental Figure S1.** *lacs4-7* is comparable to *Z377* in primary root length and lateral root density when grown on IBA.

**Supplemental Figure S2.** *lacs4* and *lacs6* are resistant to IBA in primary root elongation.

**Supplemental Table S1**. Descriptions of candidate genes mutated in *Z377* and the mutant allele tested for resistance to IBA.

**Supplementary Table S2.** Primer pairs and enzymes required for genotyping each mutant used in this study.

**Supplementary Table S3.** Sequences for new primers used in this study.

## Supplementary Material

kiaa002_Supplementary_Data

## Notes

V.J. and B.K.Z. designed the project. B.K.Z. conducted the initial screen and V.J. designed and performed further experiments. V.J. and B.K.Z. wrote the article. B.K.Z. agrees to serve as the author responsible for contact and ensures communication (zolmanb@umsl.edu).

The author responsible for distribution of materials integral to the findings presented in this article in accordance with the policy described in the Instructions for Authors (https://academic.oup.com/plphys) is: Bethany Karlin Zolman (zolmanb@umsl.edu).

## References

[kiaa002-B1] Adham AR , Zolman BK, Millius A, Bartel B (2005) Mutations in Arabidopsis acyl-CoA oxidase genes reveal distinct and overlapping roles in beta-oxidation. Plant J 41: 859–874 15743450 10.1111/j.1365-313X.2005.02343.x

[kiaa002-B2] Agrawal G , Subramani S (2016) De novo peroxisome biogenesis: evolving concepts and conundrums. Biochim Biophys Acta 1863: 892–901 26381541 10.1016/j.bbamcr.2015.09.014 PMC4791208

[kiaa002-B3] Arvidsson S , Kwasniewski M, Riaño-Pachón DM, Mueller-Roeber B (2008) QuantPrime–a flexible tool for reliable high-throughput primer design for quantitative PCR. BMC Bioinformatics 9: 465 18976492 10.1186/1471-2105-9-465 PMC2612009

[kiaa002-B4] Bartel B , Fink GR (1994) Differential regulation of an auxin-producing nitrilase gene family in Arabidopsis thaliana. Proc Natl Acad Sci USA 91: 6649–6653 8022831 10.1073/pnas.91.14.6649 PMC44260

[kiaa002-B5] Bartel B , Fink GR (1995) ILR1, an amidohydrolase that releases active indole-3-acetic acid from conjugates. Science 268: 1745–1748 7792599 10.1126/science.7792599

[kiaa002-B6] Brocard C , Hartig A (2006) Peroxisome targeting signal 1: is it really a simple tripeptide? Biochim Biophys Acta 1763: 1565–1573 17007944 10.1016/j.bbamcr.2006.08.022

[kiaa002-B7] Brown L-A , Baker A (2008) Shuttles and cycles: transport of proteins into the peroxisome matrix (review). Mol Membr Biol 25: 363–375 18651315 10.1080/09687680802130583

[kiaa002-B8] Chowdhary G , Kataya ARA, Lingner T, Reumann S (2012) Non-canonical peroxisome targeting signals: identification of novel PTS1 tripeptides and characterization of enhancer elements by computational permutation analysis. BMC Plant Biol 12: 142 22882975 10.1186/1471-2229-12-142 PMC3487989

[kiaa002-B9] Clough SJ , Bent AF (1998) Floral dip: a simplified method forAgrobacterium-mediated transformation ofArabidopsis thaliana. Plant J 16: 735–743 10069079 10.1046/j.1365-313x.1998.00343.x

[kiaa002-B10] Damodaran S , Strader LC (2019) Indole 3-butyric acid metabolism and transport in *Arabidopsis thaliana*. Front Plant Sci 10: 851 31333697 10.3389/fpls.2019.00851 PMC6616111

[kiaa002-B11] Davies RT , Goetz DH, Lasswell J, Anderson MN, Bartel B (1999) IAR3 encodes an auxin conjugate hydrolase from Arabidopsis. Plant Cell 11: 365–376 10072397 10.1105/tpc.11.3.365 PMC144182

[kiaa002-B12] De Rybel B , Audenaert D, Xuan W, Overvoorde P, Strader LC, Kepinski S, Hoye R, Brisbois R, Parizot B, Vanneste S, et al (2012) A role for the root cap in root branching revealed by the non-auxin probe naxillin. Nat Chem Biol 8: 798–805 22885787 10.1038/nchembio.1044 PMC3735367

[kiaa002-B13] Earley KW , Haag JR, Pontes O, Opper K, Juehne T, Song K, Pikaard CS (2006) Gateway-compatible vectors for plant functional genomics and proteomics. Plant J 45: 616–629 16441352 10.1111/j.1365-313X.2005.02617.x

[kiaa002-B14] Eastmond PJ , Hooks MA, Williams D, Lange P, Bechtold N, Sarrobert C, Nussaume L, Graham IA (2000) Promoter trapping of a novel medium-chain acyl-CoA oxidase, which is induced transcriptionally during Arabidopsis seed germination. J Biol Chem 275: 34375–34381 10918060 10.1074/jbc.M004945200

[kiaa002-B15] Frick EM , Strader LC (2018) Roles for IBA-derived auxin in plant development. J Exp Bot 69: 169–177 28992091 10.1093/jxb/erx298 PMC5853464

[kiaa002-B16] Friml J , Jones AR (2010) Endoplasmic reticulum: the rising compartment in auxin biology. Plant Physiol 154: 458–462 20921163 10.1104/pp.110.161380 PMC2948984

[kiaa002-B17] Fulda M (2004) Peroxisomal acyl-CoA synthetase activity is essential for seedling development in *Arabidopsis thaliana*. Plant Cell 16: 394–405 14742880 10.1105/tpc.019646 PMC341912

[kiaa002-B18] Graham IA (2008) Seed storage oil mobilization. Annu Rev Plant Biol 59: 115–142 18444898 10.1146/annurev.arplant.59.032607.092938

[kiaa002-B19] Grevengoed TJ , Klett EL, Coleman RA (2014) Acyl-CoA metabolism and partitioning. Annu Rev Nutr 34: 1–30 24819326 10.1146/annurev-nutr-071813-105541 PMC5881898

[kiaa002-B20] Groot PHE , Scholte HR, Hülsmann WC (1976) Fatty acid activation: specificity, localization, and function. Adv Lipid Res 14: 75–126 3952 10.1016/b978-0-12-024914-5.50009-7

[kiaa002-B21] Haughn GW , Somerville C (1986) Sulfonylurea-resistant mutants of *Arabidopsis thaliana*. Mol Gen Genet 204: 430–434

[kiaa002-B22] Hayashi M , Nito K, Takei-Hoshi R, Yagi M, Kondo M, Suenaga A, Yamaya T, Nishimura M (2002) Ped3p is a peroxisomal ATP-binding cassette transporter that might supply substrates for fatty acid beta-oxidation. Plant Cell Physiol 43: 1–11 11828016 10.1093/pcp/pcf023

[kiaa002-B23] Hayashi M , Toriyama K, Kondo M, Nishimura M (1998) 2,4-Dichlorophenoxybutyric acid–resistant mutants of Arabidopsis have defects in glyoxysomal fatty acid β-oxidation. Plant Cell 10: 183–195 9490742 10.1105/tpc.10.2.183 PMC143991

[kiaa002-B24] Hooper CM , Castleden IR, Tanz SK, Aryamanesh N, Millar AH (2017) SUBA4: the interactive data analysis centre for Arabidopsis subcellular protein locations. Nucleic Acids Res 45: D1064–D1074 27899614 10.1093/nar/gkw1041 PMC5210537

[kiaa002-B25] Horton P , Park K-J, Obayashi T, Fujita N, Harada H, Adams-Collier CJ, Nakai K (2007) WoLF PSORT: protein localization predictor. Nucleic Acids Res 35: W585–W597 17517783 10.1093/nar/gkm259 PMC1933216

[kiaa002-B26] Hu J , Baker A, Bartel B, Linka N, Mullen RT, Reumann S, Zolman BK (2012) Plant peroxisomes: biogenesis and function. Plant Cell 24: 2279–2303 22669882 10.1105/tpc.112.096586 PMC3406917

[kiaa002-B27] Islinger M , Li KW, Seitz J, Völkl A, Lüers GH (2009) Hitchhiking of Cu/Zn superoxide dismutase to peroxisomes—evidence for a natural piggyback import mechanism in mammals. Traffic 10: 1711–1721 19686298 10.1111/j.1600-0854.2009.00966.x

[kiaa002-B28] Jessen D , Olbrich A, Knüfer J, Krüger A, Hoppert M, Polle A, Fulda M (2011) Combined activity of LACS1 and LACS4 is required for proper pollen coat formation in Arabidopsis. Plant J 68: 715–726 21790813 10.1111/j.1365-313X.2011.04722.x

[kiaa002-B29] Jessen D , Roth C, Wiermer M, Fulda M (2015) Two activities of long-chain acyl-coenzyme A synthetase are involved in lipid trafficking between the endoplasmic reticulum and the plastid in Arabidopsis. Plant Physiol 167: 351–366 25540329 10.1104/pp.114.250365 PMC4326746

[kiaa002-B30] Kao Y-T , Gonzalez KL, Bartel B (2018) peroxisome function, biogenesis, and dynamics in plants. Plant Physiol 176: 162–177 29021223 10.1104/pp.17.01050 PMC5761812

[kiaa002-B31] Kataya ARA , Heidari B, Hagen L, Kommedal R, Slupphaug G, Lillo C (2015) Protein phosphatase 2A holoenzyme is targeted to peroxisomes by piggybacking and positively affects peroxisomal β-oxidation. Plant Physiol 167: 493–506 25489022 10.1104/pp.114.254409 PMC4326747

[kiaa002-B32] Kelley LA , Mezulis S, Yates CM, Wass MN, Sternberg MJE (2015) The Phyre2 web portal for protein modeling, prediction and analysis. Nat Protoc 10: 845–858 25950237 10.1038/nprot.2015.053 PMC5298202

[kiaa002-B33] Khan BR , Zolman BK (2010) pex5 Mutants that differentially disrupt PTS1 and PTS2 peroxisomal matrix protein import in Arabidopsis. Plant Physiol 154: 1602–1615 20974890 10.1104/pp.110.162479 PMC2996013

[kiaa002-B34] Korasick DA , Enders TA, Strader LC (2013) Auxin biosynthesis and storage forms. J Exp Bot 64: 2541–2555 23580748 10.1093/jxb/ert080 PMC3695655

[kiaa002-B35] Landrum M , Smertenko A, Edwards R, Hussey PJ, Steel PG (2010) BODIPY probes to study peroxisome dynamics in vivo. Plant J 62: 529–538 20113442 10.1111/j.1365-313X.2010.04153.x

[kiaa002-B36] Lanyon-Hogg T , Warriner SL, Baker A (2010) Getting a camel through the eye of a needle: the import of folded proteins by peroxisomes. Biol Cell 102: 245–263 20146669 10.1042/BC20090159

[kiaa002-B37] Lewin TM , Kim J-H, Granger DA, Vance JE, Coleman RA (2001) Acyl-CoA synthetase isoforms 1, 4, and 5 are present in different subcellular membranes in rat liver and can be inhibited independently. J Biol Chem 276: 24674–24679 11319232 10.1074/jbc.M102036200

[kiaa002-B38] Lewin TM , Van Horn CG, Krisans SK, Coleman RA (2002) Rat liver acyl-CoA synthetase 4 is a peripheral-membrane protein located in two distinct subcellular organelles, peroxisomes, and mitochondrial-associated membrane. Arch Biochem Biophys 404: 263–270 12147264 10.1016/s0003-9861(02)00247-3

[kiaa002-B39] Li Y , Liu Y, Zolman BK (2019) Metabolic alterations in the enoyl-CoA hydratase 2 mutant disrupt peroxisomal pathways in seedlings. Plant Physiol 180: 1860–1876 31138624 10.1104/pp.19.00300 PMC6670076

[kiaa002-B40] Lousa CDM , De Marcos Lousa C, van Roermund CWT, Postis VLG, Dietrich D, Kerr ID, Wanders RJA, Baldwin SA, Baker A, Theodoulou FL (2013) Intrinsic acyl-CoA thioesterase activity of a peroxisomal ATP binding cassette transporter is required for transport and metabolism of fatty acids. Proc Natl Acad Sci USA 110: 1279–1284 23288899 10.1073/pnas.1218034110 PMC3557071

[kiaa002-B41] Ludwig-Müller J , Epstein E (1992) Indole-3-acetic acid is converted to indole-3-butyric acid by seedlings of Zea mays L. Prog Plant Growth Regul 13: 188–193

[kiaa002-B42] Ludwig-muller J , Hilgenberg W (1995) Characterization and partial purification of indole-3-butyric acid synthetase from maize (Zea mays). Physiol Plant 94: 651–660

[kiaa002-B43] Lutz R , Bujard H (1997) Independent and tight regulation of transcriptional units in *Escherichia coli* via the LacR/O, the TetR/O and AraC/I1-I2 regulatory elements. Nucleic Acids Res 25: 1203–1210 9092630 10.1093/nar/25.6.1203 PMC146584

[kiaa002-B44] Michniewicz M , Frick EM, Strader LC (2015) Gateway-compatible tissue-specific vectors for plant transformation. BMC Res Notes 8: 63 25884475 10.1186/s13104-015-1010-6 PMC4352289

[kiaa002-B45] Middleton AM , Dal Bosco C, Chlap P, Bensch R, Harz H, Ren F, Bergmann S, Wend S, Weber W, Hayashi K-I, et al (2018) Data-driven modeling of intracellular auxin fluxes indicates a dominant role of the ER in controlling nuclear auxin uptake. Cell Rep 22: 3044–3057 29539430 10.1016/j.celrep.2018.02.074

[kiaa002-B46] Milger K , Herrmann T, Becker C, Gotthardt D, Zickwolf J, Ehehalt R, Watkins PA, Stremmel W, Fullekrug J (2006) Cellular uptake of fatty acids driven by the ER-localized acyl-CoA synthetase FATP4. J Cell Sci 119: 4678–4688 17062637 10.1242/jcs.03280

[kiaa002-B47] Mullen RT , Trelease RN (2006) The ER-peroxisome connection in plants: development of the “ER semi-autonomous peroxisome maturation and replication” model for plant peroxisome biogenesis. Biochim Biophys Acta Mol Cell Res 1763: 1655–1668 10.1016/j.bbamcr.2006.09.011 17049631

[kiaa002-B48] Neff MM , Turk E, Kalishman M (2002) Web-based primer design for single nucleotide polymorphism analysis. Trends Genet 18: 613–615 12446140 10.1016/s0168-9525(02)02820-2

[kiaa002-B49] Nyathi Y , De Marcos Lousa C, van Roermund CW, Wanders RJA, Johnson B, Baldwin SA, Theodoulou FL, Baker A (2010) The Arabidopsis peroxisomal ABC transporter, comatose, complements the *Saccharomyces cerevisiae* pxa1 pxa2Delta mutant for metabolism of long-chain fatty acids and exhibits fatty acyl-CoA-stimulated ATPase activity. J Biol Chem 285: 29892–29902 20659892 10.1074/jbc.M110.151225 PMC2943281

[kiaa002-B50] Reumann S (2004) Specification of the peroxisome targeting signals type 1 and type 2 of plant peroxisomes by bioinformatics analyses. Plant Physiol 135: 783–800 15208424 10.1104/pp.103.035584 PMC514115

[kiaa002-B51] Saryi NAA , Hutchinson JD, Al-Hejjaj MY, Sedelnikova S, Baker P, Hettema EH (2017). Pnc1 piggy-back import into peroxisomes relies on Gpd1 homodimerisation. Sci Rep 7: 42579 28209961 10.1038/srep42579 PMC5314374

[kiaa002-B52] Schwarz R , Dayhoff M (1979) Matrices for Detecting Distant Relationships. In: Dayhoff M, editor, Atlas of Protein Sequences. National Biomedical Research Foundation, Silver Springs, Maryland USA, pp. 353–358.

[kiaa002-B53] Shockey JM , Fulda MS, Browse JA (2002) Arabidopsis contains nine long-chain acyl-coenzyme a synthetase genes that participate in fatty acid and glycerolipid metabolism. Plant Physiol 129: 1710–1722 12177484 10.1104/pp.003269 PMC166759

[kiaa002-B54] Sparkes IA , Runions J, Kearns A, Hawes C (2006) Rapid, transient expression of fluorescent fusion proteins in tobacco plants and generation of stably transformed plants. Nat Protoc 1: 2019–2025 17487191 10.1038/nprot.2006.286

[kiaa002-B55] Spiess GM , Hausman A, Yu P, Cohen JD, Rampey RA, Zolman BK (2014) Auxin input pathway disruptions are mitigated by changes in auxin biosynthetic gene expression in Arabidopsis. Plant Physiol 165: 1092–1104 24891612 10.1104/pp.114.236026 PMC4081324

[kiaa002-B56] Strader LC , Bartel B (2011) Transport and metabolism of the endogenous auxin precursor indole-3-butyric acid. Mol Plant 4: 477–486 21357648 10.1093/mp/ssr006 PMC3098716

[kiaa002-B57] Strader LC , Wheeler DL, Christensen SE, Berens JC, Cohen JD, Rampey RA, Bartel B ( 2011) Multiple facets of Arabidopsis seedling development require indole-3-butyric acid-derived auxin. Plant Cell 23: 984–999 21406624 10.1105/tpc.111.083071 PMC3082277

[kiaa002-B58] Tan X-L , Xiao-li TAN, Zheng X-F, Zhang Z-Y, Wang Z, Heng-chuan XIA, Changming LU, GU Shou-lai (2014) Long chain acyl-coenzyme A synthetase 4 (BnLACS4) gene from *Brassica napus* enhances the yeast lipid contents. J Integr Agric 13: 54–62

[kiaa002-B59] Thoms S (2015) Import of proteins into peroxisomes: piggybacking to a new home away from home. Open Biol 5: 150148 26581572 10.1098/rsob.150148 PMC4680570

[kiaa002-B60] Trujillo-Hernandez JA , Bariat L, Strader LC, Reichheld J-P, Belin C (2020) A glutathione-dependent control of the indole butyric acid pathway supports Arabidopsis root system adaptation to phosphate deprivation. J Exp Bot 71: 4843–4857. 10.1093/jxb/eraa195 PMC7410191 32309856

[kiaa002-B61] Ulmasov T , Murfett J, Hagen G, Guilfoyle TJ (1997) Aux/IAA proteins repress expression of reporter genes containing natural and highly active synthetic auxin response elements. Plant Cell 9: 1963 9401121 10.1105/tpc.9.11.1963 PMC157050

[kiaa002-B62] UniProt Consortium (2019) UniProt: a worldwide hub of protein knowledge. Nucleic Acids Res 47: D506–D515 30395287 10.1093/nar/gky1049 PMC6323992

[kiaa002-B63] Wain RL , Wightman F (1954) The growth regulating activity of certain omega-substituted alkyl carboxylic acids in relation to their beta-oxidation within the plant. Proc R Soc Lond B Biol Sci 142: 525–536 13215509 10.1098/rspb.1954.0041

[kiaa002-B64] Wanders RJA , Waterham HR (2006) Biochemistry of mammalian peroxisomes revisited. Annu Rev Biochem 75: 295–332 16756494 10.1146/annurev.biochem.74.082803.133329

[kiaa002-B65] Wasternack C , Hause B (2013) Jasmonates: biosynthesis, perception, signal transduction and action in plant stress response, growth and development. An update to the 2007 review in Annals of Botany. Ann Bot 111: 1021–1058 23558912 10.1093/aob/mct067 PMC3662512

[kiaa002-B66] Watkins PA (1997) Fatty acid activation. Prog Lipid Res 36: 55–83 9373621 10.1016/s0163-7827(97)00004-0

[kiaa002-B67] Wiszniewski AAG , Zhou W, Smith SM, Bussell JD (2009) Identification of two Arabidopsis genes encoding a peroxisomal oxidoreductase-like protein and an acyl-CoA synthetase-like protein that are required for responses to pro-auxins. Plant Mol Biol 69: 503–515 19043666 10.1007/s11103-008-9431-4

[kiaa002-B68] Woodward AW , Bartel B (2005) The Arabidopsis peroxisomal targeting signal type 2 receptor PEX7 is necessary for peroxisome function and dependent on PEX5. Mol Biol Cell 16: 573–583 15548601 10.1091/mbc.E04-05-0422 PMC545895

[kiaa002-B69] Xuan W , Audenaert D, Parizot B, Möller BK, Njo MF, De Rybel B, De Rop G, Van Isterdael G, Mähönen AP, Vanneste S, et al (2015) Root cap-derived auxin pre-patterns the longitudinal axis of the Arabidopsis root. Curr Biol 25: 1381–1388 25959963 10.1016/j.cub.2015.03.046

[kiaa002-B70] Yates CM , Filippis I, Kelley LA, Sternberg MJE (2014) SuSPect: enhanced prediction of single amino acid variant (SAV) phenotype using network features. J Mol Biol 426: 2692–2701 24810707 10.1016/j.jmb.2014.04.026 PMC4087249

[kiaa002-B71] Zhao L , Haslam TM, Sonntag A, Molina I, Kunst L (2019) Functional overlap of long-chain acyl-CoA synthetases in Arabidopsis. Plant Cell Physiol 60: 1041–1054 30715495 10.1093/pcp/pcz019

[kiaa002-B72] Zolman BK , Martinez N, Millius A, Adham AR, Bartel B (2008) Identification and characterization of Arabidopsis indole-3-butyric acid response mutants defective in novel peroxisomal enzymes. Genetics 180: 237–251 18725356 10.1534/genetics.108.090399 PMC2535678

[kiaa002-B73] Zolman BK , Monroe-Augustus M, Thompson B, Hawes JW, Krukenberg KA, Matsuda SP, Bartel B (2001) chy1, an Arabidopsis mutant with impaired beta-oxidation, is defective in a peroxisomal beta-hydroxyisobutyryl-CoA hydrolase. J Biol Chem 276: 31037–31046 11404361 10.1074/jbc.M104679200

[kiaa002-B74] Zolman BK , Nyberg M, Bartel B (2007) IBR3, a novel peroxisomal acyl-CoA dehydrogenase-like protein required for indole-3-butyric acid response. Plant Mol Biol 64: 59–72 17277896 10.1007/s11103-007-9134-2

[kiaa002-B75] Zolman BK , Silva ID, Bartel B (2001) The Arabidopsis pxa1 mutant is defective in an ATP-binding cassette transporter-like protein required for peroxisomal fatty acid beta-oxidation. Plant Physiol 127: 1266–1278 11706205 PMC129294

[kiaa002-B76] Zolman BK , Yoder A, Bartel B (2000) Genetic analysis of indole-3-butyric acid responses in Arabidopsis thaliana reveals four mutant classes. Genetics 156: 1323–1337 11063705 10.1093/genetics/156.3.1323 PMC1461311

