# Supplementary material for: Long chain acyl CoA synthetase 4 catalyzes the first step in peroxisomal indole-3-butyric acid to IAA conversion
Source: Plant Physiol. 2020 Nov 17;185(1):120–36. doi: 10.1093/plphys/kiaa002 (PMC8133310; doi:10.1093/plphys/kiaa002)
Supplement: kiaa002_Supplementary_Data [file kiaa002_supplementary_data.zip › kiaa002-suppl_data/pp.00712.2020-s01.pdf]

Gene Candidates in *Z377*

ATG number	Gene name and description	Mutation tested
AT4G15280	UDP-GLUCOSYL TRANSFERASE 71B5	GK-485D08
AT4G21700	DUF2921 family protein	SAIL_172_B06
AT4G22730	Leucine-rich repeat protein kinase family protein	SALK_013143
AT4G23400	PCR55, Plasma membrane intrinsic protein	Not tested*
AT4G23850	LONG-CHAIN ACYL-COA SYNTHETASE 4, Fatty acid biosynthesis	SALK_120357
AT4G29450	Leucine-rich repeat protein kinase family protein	SAIL_337_G06
AT4G29530	Thiamine monophosphate phosphatase	SALK_101421
AT4G30190	PLASMA MEMBRANE PROTON ATPASE 2	SALK_022010
AT4G37820	Transmembrane protein; function unknown	SALK_051668

* Only available salk line contains a mutation in *ped3*, known to be resistant to IBA

Supplementary Table S1. Descriptions of candidate genes mutated in *Z377* and the mutant allele tested for resistance to IBA.

Genotyping primer pairs

Mutation	Primer Pair	Enzyme
ibr3-1	F3E22-28 & F3E22-43	EcoNI
ibr3-4	F3E22-30 & F3E22-31<br>F3E22-31 & LB-SALK	N/A
lacs4-1	LACS4-1 & LACS4-3<br>LACS4-6 & LB-SALK	N/A
lacs4-7	LACS4-5 & LACS4-7<br>LACS4-7 & LB-SALK	N/A
lacs4-8	LACS4-1 & LACS4-3	PstI-HF
lacs6-1	LACS6-1 & LACS6-4<br>LACS6-4 & LB-SALK	N/A
lacs7-1	LACS7-3 & LACS7-5<br>LACS7-5 & LB-SALK	N/A
lacs1-2	LACS1-6 & LACS1-7<br>LACS1-7 & LB-SALK	N/A
lacs2-3	LACS2-3 & LACS2-4<br>LACS2-4 & LB-GABI	N/A
lacs3-1	LACS3-1 & LACS3-2<br>LACS3-2 & LB-SALK	N/A
lacs8-2	LACS8-6 & LACS8-7<br>LACS8-7 & LB-SALK	N/A
lacs9-4	LACS9-3 & LACS9-4<br>LACS9-4 & LB-SALK	N/A

Supplementary Table S2. Primer pairs and enzymes required for genotyping each mutant used in this study.

Primer Sequences

Primer name	Sequence 5' - 3'
LACS1-6	gcaccataatgtacaccagcggcacaagc
LACS1-7	catactccatggtaatagccgacggaagcg
LACS2-3	gttgatccggtacattattactatgttatg
LACS2-4	cgatttttaaagacatgtaacttactcttc
LACS3-1	cgccttgttttagtggttagaaactccc
LACS3-2	catgtttcaggcttgcaatgctcatgg
LACS4-1	gtctttccccgaatgcagatttcaccacct
LACS4-3	gtcactatctctcataaatcacctagaatg
LACS4-5	gactttattgggcctaatatgatgatacg
LACS4-6	gagtgtgagaactgaaaagaactcaccggac
LACS4-7	catcccaacaactatccattccttcgatcg
LACS4 B	ttcggatccctaccctctggaagcaaattt
LACS4 c-term linker F	agcggcagcaagggcgaattcgacccagc
LACS4 c-term linker R	gccagacccgtaccctctggaagcaaattttg
LACS4 S	gtcgacatgtcgcagcagaagaaatac
LACS4 Topo-F	caccatgtcgcagcagaagaaatacatcttcc
LACS4 Topo-R	ctaccctctggaagcaaattttgcatttatg
LASC4 qRT F	agtacaagctgttgattccgtgtg
LACS4 qRT R	aggatatgctggtttgggttggc
LACS6-1	cttgtcttctgatcgcaatcagataatggc
LACS6-4	cagcatgcctacatagaccccaagaacag
LACS7-3	gtacacgagttcggtccgatggaaccattg
LACS7-5	gcatgatccacaaccaaccactctggtctg
LACS8-6	ggagattgtggtaggtggtaacagtgtaac
LACS8-7	cacctgtgaattgtgtgatccaggcaatg
LACS9-3	tatctcccagcaaccaagatg
LACS9-4	aagcttctccggagaattgag
UBQ10- qRT F	ttggaggatggcagaactcttgct
UBQ10-qRT R	agttttcccagtcaacgtcttaacga
YFP-SRG F	gcagaatttgaagcttgagctcgtgactaccctctggacttgtacagctcgtccatgccga
YFP-SRG R	tcggcatggacgagctgtacaagtccagagggtagtcacgagctcaagcttcaaattctgc
YFP-SRL F	ttgaagcttgagctcgtgactagagtcgggacttgtacagctcgtcc
YFP-SRL R	ggacgagctgtacaagtcccgactctagtcacgagctcaagcttcaa

Supplementary Table S3. Sequences for new primers used in this study.
