# Supplementary material for: Long chain acyl CoA synthetase 4 catalyzes the first step in peroxisomal indole-3-butyric acid to IAA conversion
Source: Plant Physiol. 2020 Nov 17;185(1):120–36. doi: 10.1093/plphys/kiaa002 (PMC8133310; doi:10.1093/plphys/kiaa002)
Supplement: kiaa002_Supplementary_Data [file kiaa002_supplementary_data.zip › kiaa002-suppl_data/pp.00712.2020-s02.pdf]

Jawahir, Supplementary Figure S1

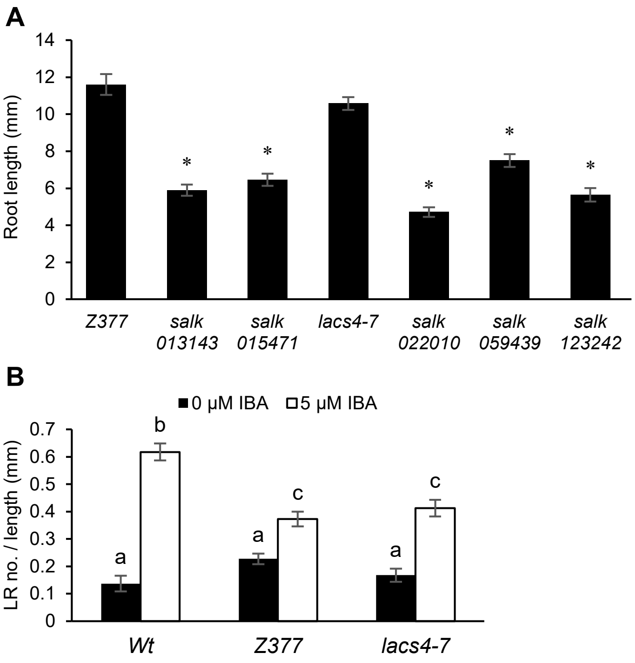

Supplemental Figure S1. *lacs4-7* is comparable to *Z377* in primary root length and lateral root density when grown on IBA.
(A) Primary root elongation of 7-d-old seedlings grown on 10μM IBA. Statistical significance determined by two-tailed t-test from *Z377* (±SE, n ≥ 14, *p<0.001).
(B) Lateral root density of 8-d-old seedlings was quantified by dividing the number of lateral roots by the primary root length. Statistical significant determined by two-way ANOVA with *post hoc* Tukey HSD (±SE, n ≥9, p <0.001).

Jawahir, Supplemental Figure S2

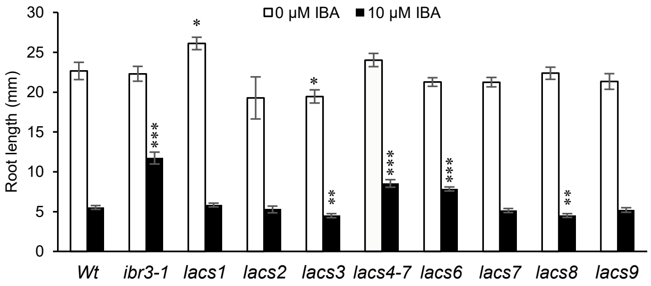

Supplemental Figure S2. *lacs4* and *lacs6* are resistant to IBA in primary root elongation. (A) Primary root elongation of 7-d-old seedlings grown on PN and PN + 10µM IBA. Statistical significance determined by two-tailed t -test compared to *Wt* of same treatment (±SE, n ≥9, *p<0.05, **p<0.01, *** p<0.001).
