# Supplementary figures and images for: Long chain acyl CoA synthetase 4 catalyzes the first step in peroxisomal indole-3-butyric acid to IAA conversion
Source: Plant Physiol. 2020 Nov 17;185(1):120–36. doi: 10.1093/plphys/kiaa002 (PMC8133310; doi:10.1093/plphys/kiaa002)

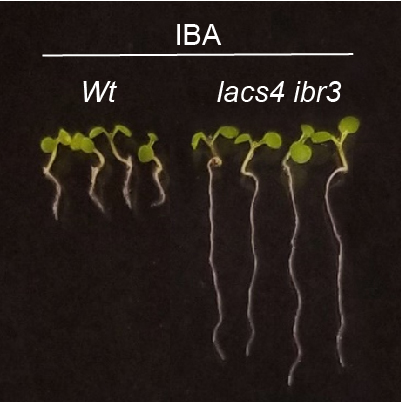

Supplement: kiaa002_Supplementary_Data [file kiaa002_supplementary_data.zip › kiaa002-suppl_data/pp.00712.2020-s03.jpg]
